# Structure-based design, synthesis, and biological evaluation of novel piperine–resveratrol hybrids as antiproliferative agents targeting SIRT-2†

**DOI:** 10.1039/d1ra04061h

**Published:** 2021-07-27

**Authors:** Ahmed H. Tantawy, Xiang-Gao Meng, Adel A. Marzouk, Ali Fouad, Ahmed H. Abdelazeem, Bahaa G. M. Youssif, Hong Jiang, Man-Qun Wang

**Affiliations:** Hubei Insect Resources Utilization and Sustainable Pest Management Key Laboratory, College of Plant Science and Technology, Huazhong Agricultural University Wuhan 430070 People's Republic of China mqwang@mail.hzau.edu.cn ahmed.tantawy@mail.hzau.edu.cn; Department of Chemistry, College of Science, Huazhong Agricultural University Wuhan 430070 China jianghong0066@126.com; Department of Chemistry, College of Science, Benha University Benha 13518 Egypt; Key Laboratory of Pesticide and Chemical Biology, Ministry of Education, School of Chemistry, Central China Normal University Wuhan 430079 China xianggao_meng@126.com; Department of Pharmaceutical Chemistry, Faculty of Pharmacy, Al-Azhar University Assiut Branch Assiut 71524 Egypt; Department of Medicinal Chemistry, Faculty of Pharmacy, Beni-Suef University Beni-Suef 62514 Egypt; Department of Pharmaceutical Sciences, College of Pharmacy, Riyadh Elm University Riyadh 11681 Saudi Arabia; Pharmaceutical Organic Chemistry Department, Faculty of Pharmacy, Assiut University Assiut 71526 Egypt

## Abstract

A series of novel piperine–resveratrol hybrids 5a–h was designed, synthesized, and structurally elucidated by IR, and ^1^H, ^13^C, and ^19^F NMR. Antiproliferative activities of 5a–h were evaluated by NCI against sixty cancer cell lines. Compound 5b, possessing resveratrol pharmacophoric phenolic moieties, showed a complete cell death against leukemia HL-60 (TB) and Breast cancer MDA-MB-468 with growth inhibition percentage of −0.49 and −2.83, respectively. In addition, 5b recorded significant activity against the other cancer cell lines with growth inhibition percentage between 80 to 95. New 5a–h hybrids were evaluated for their inhibitory activities against Sirt-1 and Sirt-2 as molecular targets for their antiproliferative action. Results showed that compounds 5a–h were more potent inhibitors of Sirt-2 than Sirt-1 at 5 μm and 50 μm. Compound 5b showed the strongest inhibition of Sirt-2 (78 ± 3% and 26 ± 3% inhibition at 50 μM and 5 μM, respectively). Investigation of intermolecular interaction *via* Hirschfeld surface analysis indicates that these close contacts are mainly ascribed to the O–H⋯O hydrogen bonding. To get insights into the Sirt-2 inhibitory mechanism, a docking study was performed where 5b was found to fit nicely inside both extended C-pocket and selectivity pocket and could compete with the substrate acyl-Lys. Another possible binding pattern showed that 5b could act by partial occlusion of the NAD^+^ C-pocket. Collectively, these findings would contribute significantly to better understanding the Sirt-2 inhibitory mechanism in order to develop a new generation of refined and selective Sirt-2 inhibitors.

## Abstract 2

A series of novel piperine–resveratrol hybrids 5a–h was designed, synthesized, and structurally elucidated by IR, and ^1^H, ^13^C, and ^19^F NMR.

## Graphical abstract

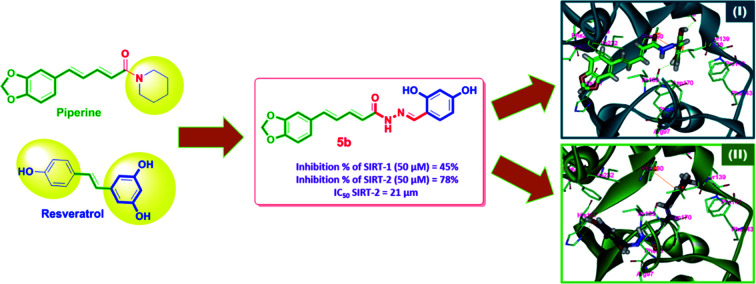

## 1. Introduction

Since cancer is one of the world's leading major health problems causing death,^1–3^ finding and discovering new successful anticancer drugs is one of the biggest challenges in drug research. Sirtuins have attracted attention over the last decade because they have participated in the regulation of many processes that affect cancer cells,^4,5^ such as cellular metabolism,^6,7^ chromatin structure control and genomic stability maintenance.^8,9^ Sirtuins are part of a family of seven human enzymes (SIRT1-7), which are NAD-related histone deacetylases (HDACs).^10,11^ With NAD, SIRTs catalyze acetyl group removal from *N*-acetyl lysine amino acid on histone substrates which generates deacetylated proteins, nicotinamides and *O*-acetyl-ADP-ribose molecules.^12,13^ Isotype Sirt-2 has taken part in several cellular processes such as gene transcription, genome constancies and cell cycle regulation during mitosis.^14,15^ Sirt-2 is a key factor in the development of cancer and metastasis by increasing cancer cell motility.^16^ In addition, Sirt-2 inhibition showed an increase in tumor suppressor genes, including p53 and p21.^17^ Small molecules that can control sirtuin activities are therefore considered potential therapeutics for the treatment of various human disorders, including cancer. Knockdown or inhibition of Sirt-2 may disrupt the metabolism of cancer cells and thus prevent the spread and growth of cancer cells.^18–21^

Natural products are one of the chief consistent sources of lead drugs. Developments based on natural products including various anticancer drugs such as topotecan, docetaxel, vindesine, etoposide and vinorelbine have been published.^22,23^ Natural products produced from bacterial, fungal, marine, plant and animal sources and natural product-inspired compounds have wide benefits in clinical trials as anti-inflammatory drugs, anticancer drugs, or other pharmaceutical agents.^24^ It is estimated that natural product-derived compounds constitute more than 50% of anticancer agents; about 74% of anticancer compounds are either natural or natural product-inspired compounds.^25^

**Fig. 1:**
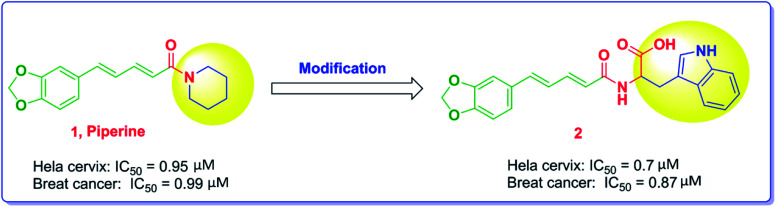
The modification of piperine structure and the impact on the anticancer activity.

Piperine, Fig. 1, is a nitrogenous alkaloid found in black pepper powder fruit that is widely used as a food flavor in a number of countries and used in many conventional food preservation systems as well as in traditional medicines.^26^ Piperine was proven to have anticancer activity with various mechanisms of action. The substitution of the piperidine moiety with bulkier and extended groups has been reported to significantly enhance the potency as noted in compound 2, where the tryptophanyl moiety has been added.^22^

**Fig. 2:**
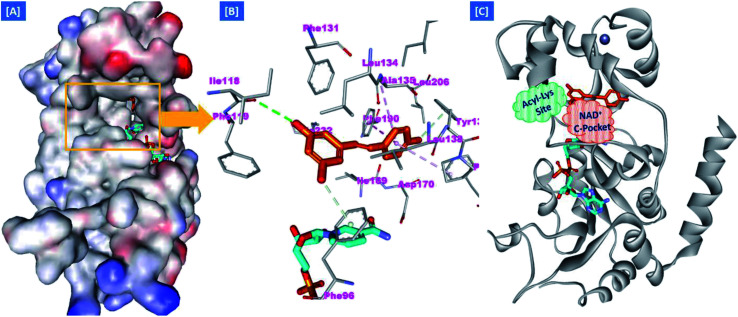
Docking of resveratrol into SIRT-2 3D structure (pdb code: 4RMG). (A) The disposition of resveratrol (orange) inside SIRT-2 active site where the protein is represented as a solid surface colored according to atom charges; (B) the predicted binding pattern and interactions of resveratrol within the active site of SIRT-2; (C) the suggested sites of structural modifications and elongation of resveratrol; green mesh represents the Acyl-Lys substrate channel and the red mesh represents the C-pocket of nicotinamide moiety of NAD^+^ co-factor (cyan). The poses were rendered as stick model and the residues are shown as smaller grey sticks. All hydrogens were removed for the purposes of clarity.

**Fig. 3:**
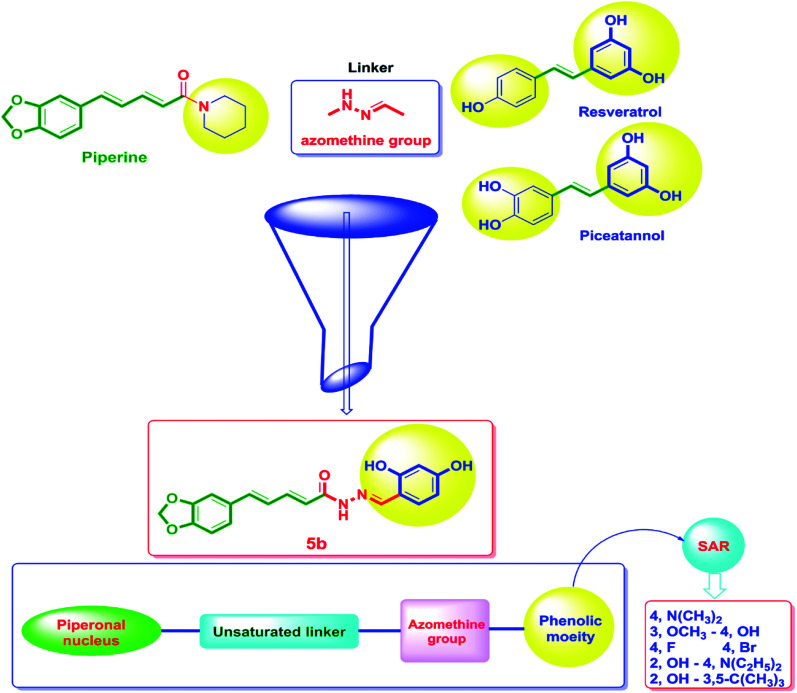
The design strategy of the novel piperine–resveratrol hybrids. In this design, the piperine backbone was tethered to 1,3-dihydroxyphenyl moiety of resveratrol or piceatannol using an azomethine group as a linker *via* a fragment-based drug design approach to obtain the final pharmacophore. Then, SAR studies was performed through replacing the phenolic moiety by with various electron-donating and electron-withdrawing substituents.

On the other hand, the polyphenolic compounds such as resveratrol and piceatannol showed high affinity to the human sirtuins family with a great modulating potential. The well-known stilbenoid polyphenol, resveratrol, was reported as a Sirt-1 and Sirt-5 activator while a weak Sirt-2 and Sirt-3 inhibitor.^27–31^ However, its metabolite piceatannol was found to act as an inhibitor to Sirt-2 protein.^31^ From the study of the reported SAR and the interactions with some SIRT proteins, it was conceptualized that the phenolic moieties have a remarkable impact on their SIRT activities.^27–31^ Till now, there are no reported studies addressing the binding pattern and the key interactions of resveratrol with Sirt-2 subtype despite the high degree of structural similarity between the conserved catalytic domains of the human sirtuins family. In order to explore this possible pattern and optimize its activity against Sirt-2 in particular, resveratrol was docked into the active site of Sirt-2 (pdb code: 4RMG) co-crystallized with the SirReal2 ligand using the Ligand Fit protocol found in Discovery Studio software 2.5. It was obvious from the docking results that resveratrol occupied a part of the extended C-site at the interface between the Rossmann-fold domain and the zinc-binding domain without any clashing with either the acyl-Lys substrate channel or the nicotinamide moiety of the co-factor NAD^+^. It was involved in only one conventional H-bond with the Ile-118 residue and some hydrophobic interactions with Leu-134 Leu-138, Tyr-139, Phe-143, and Phe-190 amino acids. This interpretation suggests a considerable possibility for further structural optimization, Fig. 2(A and B). This point can be clearly rationalized by the small size of the resveratrol structure. Inspirited by these findings, we thought that the elongation or extension of the resveratrol structure to protrude into the hydrophobic acyl-lysine tunnel surrounded by the highly conserved phenylanilines 119, 131, 234, 235 and Val233 or to partially occlude the NAD^+^ C-pocket would be a promising approach to target and competitively inhibit the Sirt-2 isoform, Fig. 2(C). Thus, our strategy to increase the size of resveratrol or even utilizing its pharmacophoric phenolic moieties was accomplished by hybridization with the piperine structure *via* a hydrazino-linker in one scaffold, Fig. 3.

Moreover, several reports showed that piperine enhanced the *in vivo* bioavailability and ADME properties of resveratrol.^32^ Hence, a commercial combination of piperine with resveratrol is currently available in the market and it is promoted to have many health benefits in terms of improving strength and endurance. Additionally, this combination is used as antioxidant, cancer protective and for weight loss. Recently, it was found that the combination of resveratrol with the polyphenolic compounds such as piperine and curcumin has a significant activity against estrogen receptor-positive MCF-7 breast cancer cells however, the authors proposed the action was accomplished through reducing glyoxalase-1 (GLO1) activity.^32,33^

Considering the aforementioned findings, a new hybrid scaffold has been designed bearing the resveratrol pharmacophoric features bound to the piperine backbone using a fragment-based drug design approach in order to target the sirtuins proteins, in particular Sirt-2, as molecular mechanism for the anticancer action of newly synthesized series, Fig. 3. To extend our study and investigate the SAR, a series of piperine derivatives were synthesized by replacement of phenolic moiety with various electron donating and electron withdrawing substituents. The identity of the newly synthesized hybrids 5a–h was proved using ^1^H, ^13^C and ^19^F NMR. Furthermore, the single-crystal structures of 5a–f were unambiguously elucidated by X-ray crystallography. The anticancer activities of 5a–h were evaluated by NCI against sixty cancer cell lines of nine different tissues.

## 2. Results and discussion

### 2.1. Chemistry

**Scheme 1:**
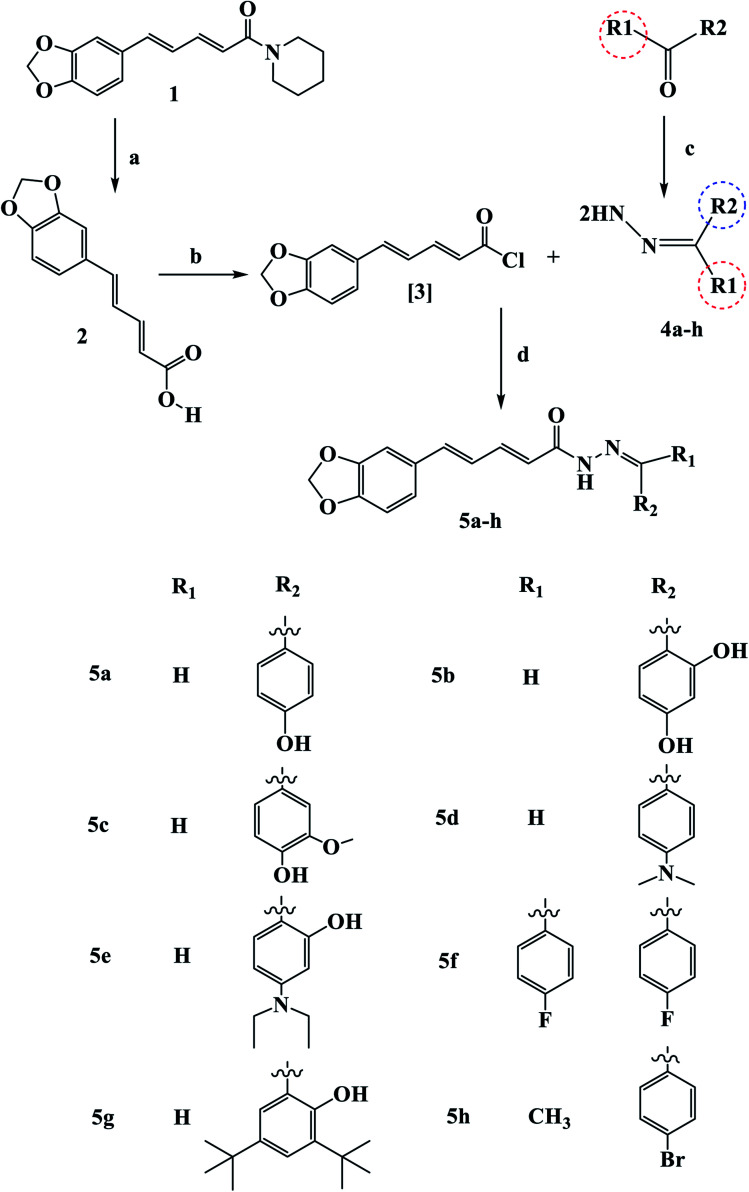
Synthetic route for the synthesis of novel piperine–resveratrol hybrids 5a–h Reagents and conditions: (a) KOH, 95% methanol, reflux, 48 h, 93%; (b) (COCl)_2_, DCM room temperature, 16 h; (c) NH_2_NH_2_, THF, reflux 24 h (d) DCM, 0 °C, 64–82%.

Scheme 1 outline the synthetic procedures for key intermediates 2, 3, and target compounds 5a–h. According to Scheme 1, piperic acid 2 was formed by hydrolyzing piperine (1) with alcoholic KOH,^34^ followed by a reaction with oxalyl chloride in the presence of DMF as a catalyst to yield piperic acid chloride 3.^35^ Carbonyl compounds were treated with hydrazine hydrate to yield the corresponding hydrazones 4a–h, which were then reacted with piperic acid chloride to yield the corresponding compounds 5a–h. The synthesized compounds were elucidated by ^1^H, ^13^C and ^19^F NMR.

The IR spectrum of 5b revealed a broad band at 3432–3300 cm^−1^ related to (OH) and strong stretching band at 1651 cm^−1^ related to (C=O), which is consistent with the proposed structure. In the ^1^H NMR of compound 5b, two common singlet signals one at 13.10–13.18 ppm related to pyridazinone ring (NHCO) and the other at 3.73–3.86 ppm associated with the benzylic protons. The ^13^C NMR spectra of Va–f showed the characteristic benzylic carbon at 35.1 ppm and the (C=O) at 168 ppm. The olefenic and aromatic carbons appeared at their expected chemical shifts.^36–38^ Interestingly, the prepared compounds are soluble in common organic solvents, such as CHCl_3_, tetrahydrofuran, and Et_2_O, and partially soluble in methanol and ethanol. Compounds 5a–f easily yielded X-ray quality crystals from slow evaporation of a mixture solution of methanol and CH_2_Cl_2_ with the appropriate amount of 5a–f at room temperature.

### 2.2. Biology

#### 2.2.1. Evaluation of *in vitro* antiproliferative activity for compounds 5a–h

The antiproliferative activity of the target compounds 5a–h was screened against a panel of 60 cancer cell lines according to NCI-guidelines at concentration of 10 μM. The results for each compound (Table 1) were recorded as the percent of growth inhibition of treated cells compared to untreated control cells. Compound 5b (**R**_**1**_**= H**, **R**_**2**_**= 2,4-dihydroxyphenyl**) was the most active among the tested compounds which directly reflects the influences of phenolic moieties on the antiproliferative activity of the title scaffold. 5b possessing a complete cell death against leukemia HL-60 (TB) and Breast cancer MDA-MB-468 with growth inhibition percentage of −0.49 and −2.83, respectively. In addition, 5b recorded significant activity against leukemia cancer cell lines K-562 and MOLT-4, Non-small cell lung cancer cell lines NCI-H322M and NCI-H460, Colon cancer cell lines COLO 205, HCT-15 and HCT-116, CNS cancer cell lines SF-295 and SF-539, Melanoma cancer cell lines LOX IMVI and UACC-62, Ovarian cancer cell lines NCI/ADR-RES, OVCAR-3, and OVCAR-4, Renal cancer cell lines CAKI-1, ACHN, and UO-31, and Breast cancer MCF7 cell line with growth inhibition percentage between 80 to 95. On the other hand, compound 5a (**R**_**1**_**= H**, **R**_**2**_**= 4-hydroxyphenyl**) showed moderate activity against the cancer cell lines studied with growth inhibition percentage between 17 to 73, Table 1. Compounds 5e (**R**_**1**_**= H**, **R**_**2**_**= 2-hydroxy-4-diethylaminophenyl**) and 5h (**R**_**1**_**= CH**_**3**_, **R**_**2**_**= 4-bromophenyl**) showed good activities against most of cancer cell lines with growth inhibition percentage between 40 to 97, Table 1. Compounds 5c, 5f and 5g bearing 3-methoxy-4-hydroxyphenyl, (bis(4-fluorophenyl)methylene) and 3,5-di-*tert*-butyl-2-hydroxyphenyl, respectively were found to be least effective against the studied cancer cell lines.

**Table 1:** One dose assay of nine different cancer cell types of compounds 5a–h^a^

Subpanel cancer cell lines	Growth% inhibition							
	5a	5b	5c	5d	5e	5f	5g	5h
**Leukemia**								
CCRF-CEM	32.40	81.02	12.13	20.48	**78.30**	—	—	44.26
HL-60(TB)	44.10	**−0.49**	—	—	**97.35**	—	—	15.88
K-562	**58.83**	**88.37**	10.48	**56.11**	**82.42**	—	—	40.06
MOLT-4	23.50	**86.66**	—	—	**81.71**	—	—	25.29
RPMI-8226	16.87	**52.34**	15.33	—	46.39	—	—	19.41
SR	**64.02**	**78.74**	—	**62.05**	**82.38**	36.26	—	**93.31**

**Non-small cell lung cancer**								
A549/ATCC	—	**63.94**	—	—	46.69	—	—	12.49
EKVX	—	**79.77**	—	—	47.83	—	—	24.99
HOP-62	—	**72.86**	—	—	**71.47**	—	—	33.23
HOP-92	—	46.60	—	—	**51.62**	13.77	—	11.51
NCI-H226	—	**62.41**	18.28	—	**50.53**	—	10.06	24.10
NCI-H23	—	**70.00**	—	—	**50.33**	—	—	—
NCI-H322M	18.48	**82.25**	14.74	—	**66.83**	—	—	36.51
NCI-H460	—	**86.03**	—	—	**80.25**	—	—	**60.40**
NCI-H522	22.76	**78.87**	21.98	11.67	**87.48**	—	—	19.91

**Colon cancer**								
COLO 205	—	**86.03**	—	—	**60.08**	—	—	—
HCC-2998	—	**61.09**	—	—	45.98	—	—	10.78
HCT-116	—	**91.33**	—	—	**92.14**	—	—	12.34
HCT-15	39.67	**83.03**	—	24.23	**78.73**	—	—	36.30
HT29	—	**51.91**	—	—	32.24	—	—	—
KM12	33.35	**76.61**	—	14.25	**69.83**	—	—	24.89
SW-620	19.77	**73.5**	—	—	**67.68**	—	—	25.44

**CNS cancer**								
SF-268	17.15	**75.57**	—	—	**66.32**	—	—	32.46
SF-295	—	**86.23**	—	—	**58.30**	—	—	—
SF-539	17.56	**85.66**	15.33	—	**77.28**	—	—	12.47
SNB-19	—	**50.87**	—	—	38.99	—	—	14.48
SNB-75	26.97	35.98	—	25.68	41.19	16.64	32.95	**68.09**
U251	—	**68.74**	—	—	**56.75**	—	—	16.04

**Melanoma**								
LOX IMVI	38.53	**90.31**	—	11.11	**81.77**	—	12.05	38.29
MALME-3M	—	**59.69**	—	—	43.47	—	—	28.67
M14	17.10	**69.73**	—	22.24	**63.64**	—	—	26.74
MDA-MB-435	73.02	**67.38**	—	**70.14**	**51.12**	—	—	13.60
SK-MEL-2	—	47.24	—	—	34.33	—	—	11.01
SK-MEL-28	—	**57.87**	—	—	47.95	—	—	14.25
UACC-257	—	**71.52**	—	—	**75.82**	—	—	12.00
UACC-62	28.50	**85.74**	11.32	16.06	**70.87**	12.19	—	28.51

**Ovarian cancer**								
IGROV1	24.72	**71.10**	—	10.14	**61.80**	12.09	14.13	29.67
OVCAR-3	—	**90.27**	—	—	**95.06**	—	—	24.42
OVCAR-4	12.67	**90.76**	—	—	**84.74**	—	—	65.24
OVCAR-5	—	**57.55**	—	—	39.11	—	—	10.69
OVCAR-8	—	**77.21**	—	—	**67.09**	—	—	24.67
NCI/ADR-RES	—	**94.85**	—	—	**82.45**	—	—	33.86
SK-OV-3	—	**73.00**	—	—	**60.49**	—	—	—

**Renal cancer**								
786-0	—	**70.98**	—	—	**52.78**	—	—	14.63
ACHN	—	**85.61**	—	—	**74.37**	—	—	24.09
CAKI-1	25.08	**90.35**	—	24.55	**70.70**	12.79	—	28.09
RXF 393	—	42.15	—	—	27.41	—	—	—
SN12C	—	**67.24**	—	—	45.91	—	—	12.40
TK-10	—	**69.04**	—	—	**62.17**	—	—	—
UO-31	39.21	**88.57**	14.03	23.71	**75.03**	18.30	27.88	44.91

**Prostate cancer**								
PC-3	19.78	**62.05**	—	15.03	**59.10**	—	—	30.92
DU-145	—	**50.01**	—	—	46.82	—	—	43.99

**Breast cance**r								
MCF7	28.77	**85.87**	20.13	43.56	**80.48**	—	—	38.66
MDA-MB-231/ATCC	17.21	**65.61**	—	—	**56.75**	—	—	21.03
HS 578 T	—	29.82	—	—	31.90	—	—	**86.45**
BT-549	21.48	**72.73**	13.62	—	48.23	—	—	—
T-47D	18.59	**76.14**	19.80	—	**73.68**	—	—	**65.01**
MDA-MB-468	—	**−2.83**	—	—	**86.64**	—	—	29.28

a (−): complete cell death, (—): not calculated.

#### 2.2.2. Sirtuins inhibitory activity of 5a–h hybrids

Sirtuin-TK assay was performed to estimate the Sirtuins inhibitory potential of 5a–h (Tables 2 and 3). The results of this test strongly complemented the findings of cancer cell-based assessments. Generally, the piperine–resveratrol 5a–h hybrids were found to be stronger inhibitors of Sirt2 than Sirt1 at 5 μM and 50 μM. Of the 8 compounds evaluated, 3 analogs (5b, 5e and 5h) were more likely to inhibit Sirt2 (>70% inhibition) than Sirt1 (<50% inhibition) at 50 μM. The other compounds were poor inhibitors of both enzymes. Of these compounds studied, 5b (**R**_**1**_**= H**, **R**_**2**_**= 2,4-dihydroxyphenyl**) showed a potent inhibition of SIRT2 (78 ± 3% and 26 ± 3% inhibition at 50 μM and 5 μM, respectively). Compared with compound 5b, which has 2,4-di-hydroxyphenyl moiety, compound 5a, containing 4-hydroxyphenyl moiety, showed lower potency to inhibit SIRT2 (72 ± 3% and 19 ± 3% inhibition at 50 μM and 5 μM, respectively). Replacement of the 4-hydroxy group in compound 5b with 4-diethylamino in compound 5e resulted in a slight reduction of the inhibitory SIRT2 values (74 ± 2% and 24 ± 3% inhibition at 50 μM and 5 μM, respectively). A further analysis of the compound pairs [5b*vs.*5c] showed that 5c (containing 4-hydroxy-3-methoxyphenyl) would likely have less potent SIRT2 inhibition (58 ± 3% and 17 ± 3% inhibition at 50 μM and 5 μM, respectively) than compound 5b (Table 3). Notably, 5h, containing 4-bromophenyl moiety, showed promising potency with 75 ± 3% and 22 ± 5% SIRT2 inhibition at 50 μM and 5 μM, respectively.

**Table 2:** Inhibitory activities of 5a–h against human SIRT1

Compound	% inhibition of SIRT1	
	5 μm	50 μm
5a	13 ± 3	42 ± 3
5b	18 ± 3	45 ± 3
5c	12 ± 1	38 ± 3
5d	11 ± 2	48 ± 3
5e	20 ± 5	44 ± 2
5f	14 ± 4	39 ± 3
5g	9 ± 2	35 ± 1
5h	16 ± 5	44 ± 3

**Table 3:** Inhibitory activities of 5a–h against human SIRT2

Compound	% inhibition of SIRT2	
	5 μm	50 μm
5a	19 ± 3	72 ± 3
5b	26 ± 3	78 ± 3
5c	17 ± 3	58 ± 3
5d	18 ± 2	70 ± 3
5e	24 ± 3	74 ± 2
5f	20 ± 4	69 ± 3
5g	19 ± 2	55 ± 1
5h	22 ± 5	75 ± 3

We then evaluated the 5b, 5e and 5h IC_50_ values against SIRT2, all of which inhibited SIRT2 by more than 75% at 50 μM compared to the SIRT2 selective inhibitor AGK2 as a reference. The results are listed in Table 4. Compound 5b was the most potent one among the tested derivatives, with IC_50_ value of 21 ± 3 μM in comparison to the reference AGK2 (IC_50_ = 13.9 ± 1 μM).^39^

**Table 4:** IC_50_ values for the inhibitory activity of compounds 5b, 5e and 5h against SIRT2 enzyme

Compound	IC_50_ (μm)
5b	21 ± 3
5e	23 ± 2
5h	26 ± 3
AGK2 (ref. 39)	13.9 ± 1

#### 2.2.3. Cell cycle analysis and apoptosis assay

##### 2.2.3.1. Cell cycle analysis

**Fig. 4:**
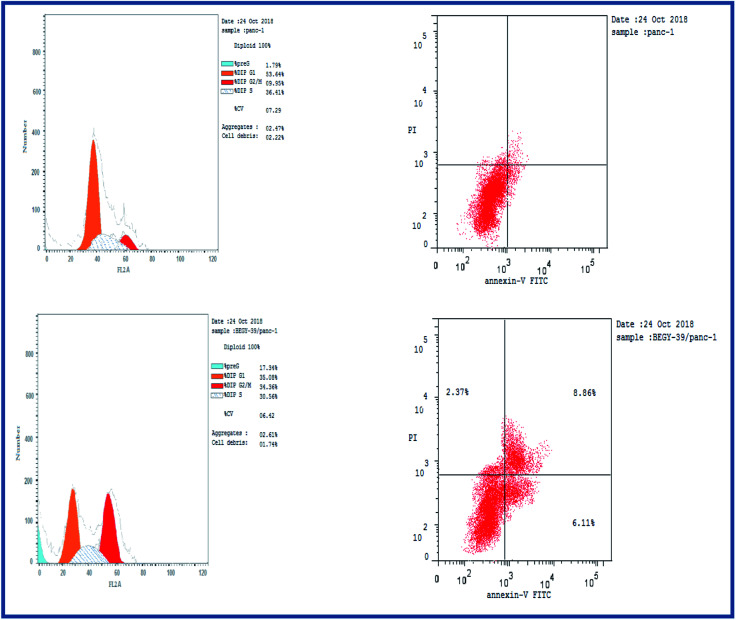
Cell cycle analysis of 5b in human pancreatic cancer cell line MCF-7.

Cell cycle analysis was conducted for the most active compound 5b against human pancreatic cancer cell line MCF-7. The percentage of MCF-7 cells in the G0/G1 phase of the control cell cycle was 53.64%, with a significant decrease to 35.08% following treatment with compound 5b, while the percentage of cells in the S phase was marginally reduced with compound 5b (35.56%) compared to the control (36.41%) (Fig. 4). The percentage of MCF-7 human pancreatic cancer cell line in the G2/M phase increased to 34.36% following treatment with compound 5b. In addition, it is clear that the percentage of apoptotic cells in the pre-G1 process increased from 1.79% for control of untreated MCF-7 human pancreatic cancer cells to 17.34% and 22.17% for controlled 5b and doxorubicin cells, respectively (Fig. 4, Table 5). According to the above results, compound 5b exhibited mainly cell cycle arrest during the Pre-G1 and G2/M phases. In addition, it is clear that the compound studied is not cytotoxic but antiproliferative, triggering programmed cell death and cell cycle arrest.

**Table 5:** Apoptosis induction analysis of compound 5b

Sample code	Cell line	Apoptosis			% necrosis
		% total	% early	% late	
5b	MCF-7	6.16	2.74	2.29	1.13
Control	MCF-7	1.72	0.88	0.23	0.61

### 2.3. Description of crystals 5a–f and Hirschfeld surface analysis

The Hirschfeld surfaces and fingerprint plots can be utilized to identify a type and region of intermolecular interactions, which are capable of being generated using Crystal-Explorer software.^40^ Molecular Hirschfeld surface in a crystal structure is constructed based on the electron distribution. Its normalized contact distance (*d*_norm_) based on both *d*_e_, *d*_i_ and van der Waals radii of the atom is listed in the following equation. Then, intermolecular contacts in the crystal can be analyzed by a combination of *d*_e_ and *d*_i_ in the form of a 2D fingerprint plot as listed in the below equation.^41^ Complementary regions are visible in the fingerprint plots where one molecule acts as a donor (*d*_e_ > *d*_i_) and the other as an acceptor (*d*_e_ < *d*_i_). The fingerprint plots can be divided into highlighting particular close interactions between the two atoms.^42^ This decomposition enables the separation of contributions from different interaction types in the full fingerprint.*d*_norm_= (*d*_i_– *r*^vdw^_i_)/*r*^vdw^_i_ + (*d*_e_ − *r*^vdw^_e_)/*r*^vdw^_e_

**Fig. 5:**
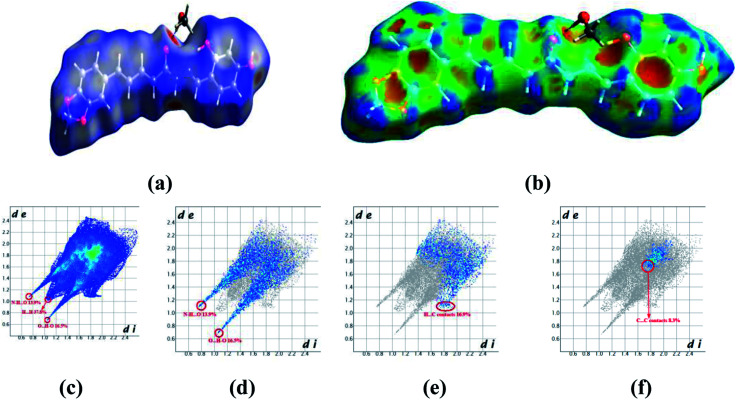
Hirshfeld surfaces in 5b mapped with (a) *d*_norm_; (b) shape index, (c) full fingerprint plot; (d) H⋯O contacts (O–H⋯O and N–H⋯O); (e) H⋯C contacts (C–H⋯π) and (f) C⋯C contacts (π⋯π).

Crystals of 5b were determined at room temperature. X-ray experiments indicate that there are each one complete 5b and one methanol solvent molecule in its asymmetric unit. The whole molecule shows a nearly flat configuration due to these several double bonds. The Hirschfeld surfaces of compound 5b have been mapped over *d*_norm_ (Fig. 5a) and shape index (Fig. 5b). The intermolecular interactions mainly originated from hydroxyl oxygen and acyl hydrazone nitrogen atoms can be seen in the Hirschfeld surface as the bright red areas in Fig. 5a, and the light red spots are corresponding to C–H⋯O and C–H⋯π interactions. The O⋯H/H⋯O intermolecular interactions (30.4%) appear as distinct spikes in the 2D fingerprint plot (Fig. 5d). In the fingerprint plots, there are two sharp spikes in the lower left of the plots due to the O/N–H⋯O hydrogen bonds (Fig. 5c). The proportion of O⋯H/H⋯O interactions comprises 30.4% of the total Hirschfeld surfaces. The points in the (*d*_i_, *d*_e_) regions around (1.168, 1.107) from the fingerprint plots belong to C–H⋯π interactions (17.2%) (Fig. 5e) which is mainly existing between the benzene ring and the methanol solvent at (2 − *x*, 1 − *y*, *z*). π⋯π interactions are not represented because there are no typical ‘wings’ at the top left and bottom right of the two-dimensional fingerprint plot (Fig. 5f), occupying *ca.* 8.3% of the total Hirschfeld surface. For the crystal packing, the molecules of 5b are linked by these O/N–H⋯O hydrogen bonds into a two-dimensional layer structure parallel to the (001) plane. These (001) layer structures are further linked into a three-dimensional network.

In the crystal structure of 5a (Fig. S9†), there is one complete molecule and one methanol molecule in its asymmetric unit in which the component ions are linked into a one-dimensional hydrogen-bonded chain running along the [010] axis. A Hirschfeld surface analysis indicates that the H⋯O (25.6%), H⋯C (22.1%), C⋯C (5.9%) and H⋯H (38.7%) contacts in 5a are comparable to those in crystal structure of 5b.

In the crystal structure of 5c, there is one complete molecule in its asymmetric unit in which the component ions are linked into a two-dimensional hydrogen-bonded layer structure parallel to the plane (20−1). A Hirschfeld surface analysis indicates that the contacts of H⋯O, H⋯C, C⋯C and H⋯H in the crystal packing of 5c are 22.8%, 28.8%,1.4% and 35.3% respectively (Fig. S10†). In 5d, there is one complete molecule and two water molecules in its asymmetric unit in which the component ions are linked into a two-dimensional hydrogen-bonded network parallel to the (001) plane. A Hirschfeld surface analysis for the host molecule indicates that the H⋯O, H⋯C and C⋯C contacts including their reciprocal contacts are 19.2%, 27.1% and 3.2% of the total surface, respectively (Fig. S11†).

In 5e, there is each one host molecule and one methanol molecule in its asymmetric unit. Analysis indicates the component ions are firstly linked into a one-dimensional hydrogen-bonded chain running along the [010] axis. These neighboring [010] chains are linked into a three-dimensional network by a combination of C–H⋯π and π⋯π interactions. A Hirschfeld surface analysis indicates that the H⋯O (19.7%), H⋯C (17.4%), C⋯C (5.4%) and H⋯H (51.5%) contacts are also comparable to those in crystal structures above mentioned (Fig. S12†). In the crystal of 5f (Fig. S13†), a dimer is formed by a pair of complementary N–H⋯O hydrogen bonds. A Hirschfeld surface analysis indicates that the H⋯O (including the reciprocal contacts) contacts comprise 13.9% of the total surface. The H⋯C (C–H⋯π, 18.9%) and C⋯C (π⋯π, 5.3%) are both comparable to those in 5b.

From the Hirschfeld analysis, we can obviously see that the O/N–H⋯O hydrogen bonds are the main driving forces in the crystal packing. The C–H⋯π interactions also play an important role in the crystal aggregates. However, the π⋯π interactions comprise the least of the total Hirschfeld surface in these five structures. We can also find that the lateral hydroxyl oxygen and acyl hydrazone nitrogen atoms are in favor of hydrogen-bonding to some other acceptors. To sum up above mentioned discussion, one can find that the O/N–H⋯O hydrogen bonds are preferred in the crystallization state. Although a crystallization state cannot be completely a representation of a solution state, it can still give us some hints when these molecules are used an antiproliferative inhibitors they may firstly interact with the target position of a protein through hydrogen bonds (*vide infra*).

### 2.4. Molecular docking study

**Fig. 6:**
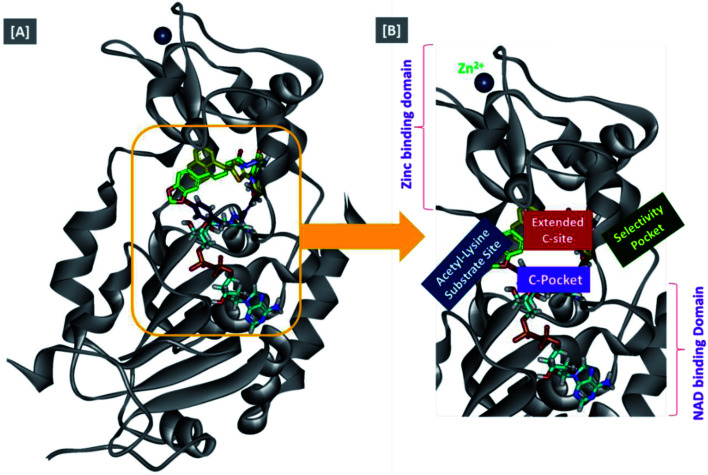
Docking of compound 5b into SIRT-2 3D structure (pdb code: 4RMG). (A) Overlay of the top docked poses 5b (green and violet) and SirReal-2 (yellow) as a co-crystallized ligand into the Sirt-2 binding site where the Sirt-2 protein is represented as secondary structure displayed in a flat ribbon style (cartoon) colored in black-white; (B) labelling of the different binding pockets of Sirt-2 protein. The NAD^+^ co-factor (cyan) is displayed in stick.

In an attempt to rationalize the obtained *in vitro* Sirt-2 assay results, a molecular docking study of the most potent compound 5b into the active site of human Sirt-2 was performed to elucidate the possible underlying inhibitory mechanisms and to predict the binding mode and the interactions that can be formed. The 3D crystal structure of Sirt-2 (PDB code: 4RMG) in complex with SirReal2 ligand and Co-factor NAD^+^ was used after preparation. The docking simulation was carried out using Ligand Fit embedded in Discovery Studio software 2.5 (San Diego, USA) according the reported method.^43–46^ Analysis of the top-ranked pose of compound 5b demonstrated several plausible molecular interactions and various binding patterns, Fig. 6A. It was reported that the active site of Sirt-2 could be divided into several sites. The C-pocket accommodates the nicotinamide moiety of the co-factor NAD^+^ while the acetyl-Lys substrate binding channel is formed by several hydrophobic phenylalanine's. In addition, there is a pocket close to the C-pocket and link the inducible selectivity pocket in Sirt-2 with the acyl-Lys tunnel called extended C-site, Fig. 6(B).^47,48^

**Fig. 7:**
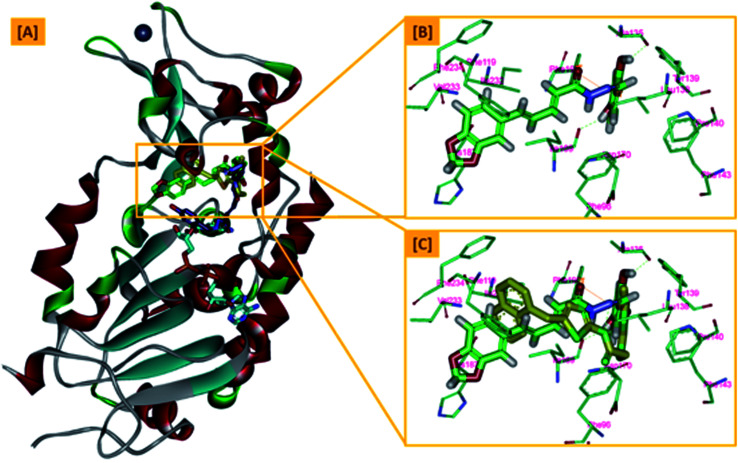
The first hypothesis of binding pattern of 5b inside Sirt-2 active site; (A) overlay of the top docked poses 5b (green and violet) and SirReal-2 (yellow) as a co-crystallized ligand into the secondary structure of Sirt-2 active pocket; (B) the interactions and binding mode of 5b (green) inside the Sirt-2 active sites (extended C-pocket and selectivity pocket); (C) superimposition of 5b (green) and SirReal2 (yellow). The poses were rendered as stick model. π–π interactions were represented as orange solid line. Hydrogen bonds were represented as dashed green lines.

**Fig. 8:**
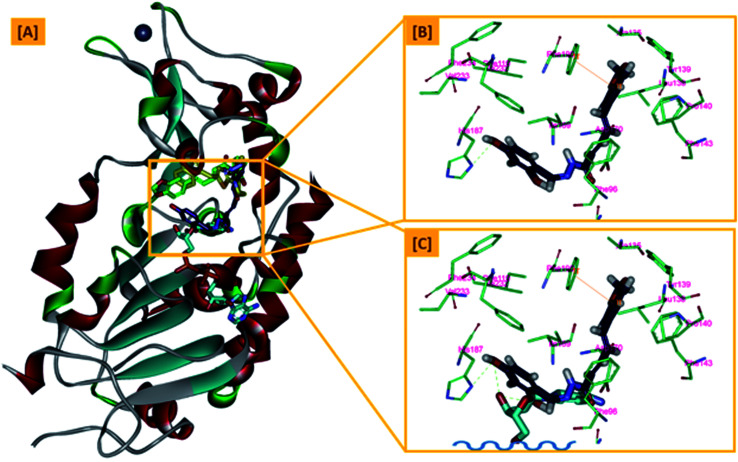
The second hypothesis of binding pattern of 5b inside Sirt-2 active site; (A) overlay of the top docked poses 5b (green and violet) and SirReal-2 (yellow) as a co-crystallized ligand into the secondary structure of Sirt-2 active pocket; (B) the interactions and binding mode of 5b (violet) inside the Sirt-2 active sites (Extended C-pocket and selectivity pocket); (C) the window shows magnification of the possible clash between 5b (violet) and the NAD^+^ co-factor (cyan). The rest of NAD^+^ was removed for clarity. The poses were rendered as stick model. π–π interactions were represented as orange solid line. Hydrogen bonds were represented as dashed green lines.

From a precise inspection of the results, it was conceptualized that there are two possible different hypotheses or binding patterns can be used to rationalize the inhibitory activity of our compound 5b against SIRT-2: (I) our newly synthesized hybrid molecule 5b adopts a very similar binding mode consistent with that of the co-crystallized ligand, SirReal2 where it binds to the extended C-site as well as to the reported selectivity pocket that formed by two loops of the hinge region connecting the Rossmann-fold and zinc-binding domains. The binding pattern analysis revealed that 1,3-dihydroxyphenyl moiety of 5b can be accommodated into the lipophilic selectivity pocket in a manner similar to the dimethyl mercapto-pyrimidine residue of SirReal2 maintaining the same π–π stacking with Phe190. In addition, two hydrogen bonds were formed between the two hydroxyl groups of 5b and Ala135 and Ile169 residues. The rest of the 5b kinked conformation occupies the Extended C-site adjacent to the C-pocket and it is oriented towards the acetyl-lysine substrate tunnel. The benzodioxolyl moiety of 5b protrudes into the substrate channel forming hydrophobic interactions with Phe119, Phe131, Ile232, Val233 and Phe234 amino acids. This bulky benzodioxolyl moiety equivalent to the naphthyl moiety of SirReal2, is thought to force the acetyl-lysine out of its physiological position in a competitive inhibitory manner. Interestingly, the 5b structure is longer than sirReal2 one which in turn will likely compete with the acyl-Lys substrate in a stronger way. In this hypothesis, 5b does not interfere with the C-pocket where nicotinamide moiety of NAD^+^ binds, Fig. 7A–C.

(II) Another considerable binding mode was observed where 5b binds in an inverted fashion to what was adopted in the former hypothesis. It was found that the benzodioxolyl moiety induces the formation of the selectivity pocket instead of the dihydroxyphenyl in a similar manner to that of sirReal2 forming the reported π–π stacking with Phe190. However, the dihydroxyphenyl moiety was oriented downwards toward the C-pocket where the nicotinamide moiety of NAD^+^ binds and initializes the deacetylation reaction. This conformation is stabilized by forming a hydrogen bond with His187. Another hydrophobic interaction between the unsaturated alkenyl chain of 5b and the gate keeper, Phe96 residue, which helps nicotinamide to release and prevents the backward reaction was observed. It was also noted that 5b was highly distorted from the extended C-site or the substrate tunnel and did not have strong hydrophobic contact with Phe119, Phe131, Ile232, Val233 and Phe234 amino acids residue, Fig. 8A–C. This hypothesis suggested that 5b might partially occupy the C-pocket and occlude or compete with nicotinamide moiety of NAD^+^ in contrary to the sirReal2 mechanism which a is partially non-competitive towards NAD^+^. We think that the former hypothesis is a much more reasonable explanation for the inhibitory activity of 5b against Sirt-2 where it shows the highest ranked binding pattern with the lowest energy score. Moreover, targeting both selectivity pocket and the substrate channel by linking two distinct moieties in one scaffold is much more acceptable and significant mechanism for inhibition of Sirt-2. Indeed, we will need further kinetic competition studies to distinguish and prove one of these two inhibitory mechanisms. However, these results support the hypothesis that targeting either the acyl-Lys substrate binding site or NAD^+^ co-factor C-pocket represents a useful approach to develop novel and potent SIRT2 competitive inhibitors.

## 3. Conclusion

In this study, a series of piperine–resveratrol hybrids 5a–h was designed and synthesized as inhibitors of Sirt-2. A panel with sixty cancer cell lines of nine different tissues in compliance with the NCI protocol has been utilized for the evaluation of 5a–h hybrids. Compounds 5b, 5e and 5h showed significant antiproliferative activity. 5a–h were found to be more potent inhibitors of Sirt-2 than Sirt-1. Results of cell cycle investigation showed that 3.64% of pre-G1 apoptosis was induced by compound 5b on MCF-7 with a high percentage of cell accumulation in G2-M phase. In these complexes with the hydroxyl groups, the component ions are mainly linked into a 3D framework by a combination of O–H⋯O hydrogen bonds, C–H⋯O, C–H⋯π and π⋯π interactions. Hirschfeld surface analysis indicates that O–H⋯O hydrogen bonds consist of *ca.* 20% of the total surface, which indicate that in the biological system the O–H⋯O interaction may be mainly attributed to their biological characteristics. A docking study of the most active compound 5b was carried out to get insights into the Sirt-2 inhibitory mechanism and justify the *in vitro* results. The results revealed two possible binding patterns of 5b that could be used to rationalize its Sirt-2 inhibitory activity by either competition with acyl-Lys substrate or partially blocking co-factor NAD^+^ C-pocket. These findings highlight the importance of Sirt-2 as a promising anticancer target and open a new avenue to develop novel superior and selective Sirt-2 inhibitors.

## 4. Experimental

### 4.1. Chemistry

General details and crystallographic data (Table S1): see ESI† and Appendix A.

#### 4.1.1. Synthesis of (2*E*,4*E*)-5-(benzo[*d*][1,3]dioxol-5-yl)penta-2,4-dienoyl chloride (3)

Compounds 2 and 3 were prepared according to our previous research work^35^ and the experimental details are recorded in Appendix A.

#### 4.1.2. Synthesis of hydrazone derivatives (4a–h)^49–51^

To a solution of carbonyl compounds (2.5 mmol), hydrazine monohydrate (158 μL, 3.25 mmol) in THF solution was added and refluxed for 24 h. Then the hydrazone solution was dried by 200 mg anhydrous Na_2_SO_4_ for 0.5 h and then 5 Å molecular sieves powder (250 mg) for 5–6 h. The obtained hydrazones 4a–h were utilized directly to the next step.

#### 4.1.3. General procedure for synthesis of piperine-based hydrazone derivatives (5a–h)

Compound 3 (191 mg, 1.1 mmol) was dissolved in CH_2_Cl_2_ (5 mL) and was added dropwise into a mixture of the hydrazones (4a–h, 100 mg, 1 mmol) in CH_2_Cl_2_ (5 mL) in ice bath. After addition of compound 3, the reaction mixture was stirred for 7 h at room temperature, and then the solvent was removed under reduced pressure. The crud product was treated with Na_2_CO_3_ solution, then extracted three times by ethyl acetate. The organic layer was dried over Na_2_SO_4_ and the solvent was evaporated under reduced pressure. The residue was purified by silica gel column chromatography (CH_2_Cl_2_ : MeOH = 20 : 1) to give the corresponding compounds 5a–h (69.1–89.2% yields). Crystals were grown after slow evaporation of a mixture solution of methanol and CH_2_Cl_2_ (2 : 3) with the appropriate amount of 5a–f, then left open to the atmosphere at room temperature, producing yellow sheets and cubic crystals after 45 days.

##### 4.1.3.1. (2*E*,4*E*)-5-(Benzo[*d*][1,3]dioxol-5-yl)-*N*′-((*E*,*Z*)-4-hydroxybenzylidene)penta-2,4-dienehydrazide (5a)

**Fig. 9:**
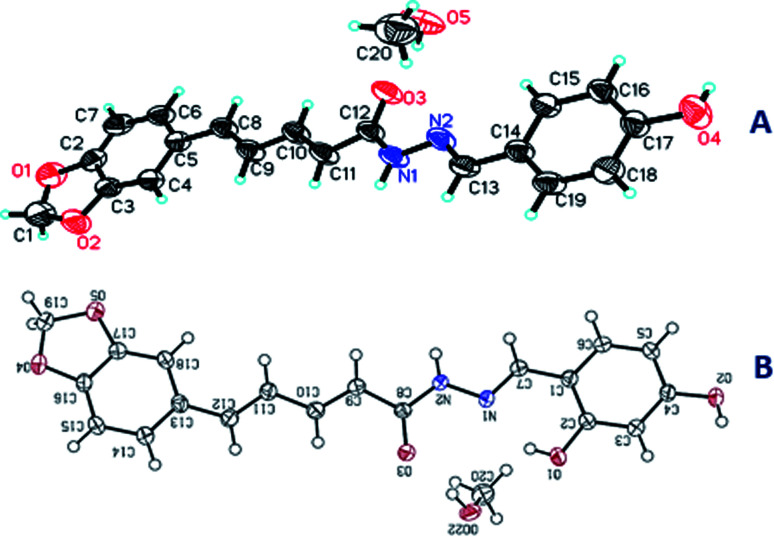
The X-ray single crystal structures of 5a (A) and 5b (B).

Pale yellow solid, m.p. 12–127 °C, yield: 83%; IR: 3463, 3104 3050, 2890, 1660, 1600, 1500, 1448, 1400, 1384, 1260. ^1^H NMR (600 MHz, DMSO-*d6*) *δ* 11.40 (s, 0.49H, NH), 11.20 (s, 0.46H, NH), 9.98 (d, 0.51H, OH), 9.91 (d, 0.46H, OH), 8.12 (s, 0.51H, 1H, CH=N), 7.93 (s, 0.46H, 1H, CH=N), 7.54 (d, *J* = 8.6 Hz, 2H), 7.41–7.26 (m, 2H), 7.20–7.07 (m, 1H), 7.02 (dd, *J* = 15.7, 6.3 Hz, 1H), 6.99–6.95 (m, 1H), 6.93 (dd, *J* = 8.0, 4.3 Hz, 1H), 6.83 (d, *J* = 8.6 Hz, 2H), 6.15 (d, *J* = 14.9 Hz, 1H), 6.06 (d, *J* = 2.8 Hz, 2H, –OCH_2_O−). ^13^C NMR (150 MHz, DMSO-*d6*) *δ* 166.47, 161.91, 159.81, 159.57, 148.45, 148.37, 147.12, 131.31, 131.25, 129.34, 129.21, 128.93, 128.79, 125.82, 123.33, 116.23, 116.04, 109.02, 106.23, 106.07, 101.79. C_20_H_20_N_2_O_5_, crystal dimensions 0.12 × 0.1 × 0.1 mm^3^, *M*_r_ = 368.38, monoclinic, space group *P*2_1_/*n* (14) cell: *a* = 10.9249(5), *b* = 6.5740(3), *c* = 25.7341(11) Å, *α* = 90°, *β* = 94.790(3)°, *γ* = 90°, *V* = 1841.78(14) Å^3^, *Z* = 4, density (calculated) = 1.329 g m^−3^, *μ* = 0.799 mm^−1^, *F*(000) = 776.0, reflection collected/unique = 10 352/3035, refinement method = full-matrix least-squares on *F*2, Final *R* indices [*I* > 2sigma(*I*)]: *R*_1_ = 0.0925, w*R*_2_ = 0.2368, *R* indices (all data): *R*_1_ = 0.1011, w*R*_2_ = 0.2530, goodness of fit on *F*^2^ = 1.026. CCDC 1984251 (Fig. 9A).

##### 4.1.3.2. (2*E*,4*E*)-5-(Benzo[*d*][1,3]dioxol-5-yl)-*N*′-((*E*)-2,4-dihydroxybenzylidene)penta-2,4-dienehydrazide (5b)

Yellow solid, m.p. 153–156 °C, yield: 80%; IR: 3432, 3300, 3030, 2902, 1650, 1602, 1515, 1439, 1367, 1252. ^1^H NMR (600 MHz, DMSO-*d6*) *δ* 11.64 (s, 1H, NH), 11.38 (s, 1H, OH), 9.95 (s, 1H, OH), 8.24 (d, *J* = 4.8 Hz, 1H, CH=N), 7.38–7.31 (m, 1H), 7.29 (d, *J* = 7.8 Hz, 2H), 7.07–6.96 (m, 3H), 6.93 (t, *J* = 8.2 Hz, 1H), 6.37–6.28 (m, 2H), 6.14 (d, *J* = 14.9 Hz, 1H), 6.06 (s, 2H, –OCH_2_O−). ^13^C NMR (151 MHz, DMSO-*d6*) *δ* 166.4, 164.1, 161.9, 150.3, 148.5, 142.5, 141.5, 140.9, 140.1, 136.0, 131.2, 126.2, 125.9, 125.6, 123.9, 123.6, 121.9, 117.6, 114.9, 113.2, 111.1, 109.0, 106.4, 102.0. C_20_H_20_N_2_O_6_, crystal dimensions 0.12 × 0.1 × 0.1 mm^3^, *M*_r_ = 384.38, monoclinic, space group *P*2_1_/*c*(14) cell: *a* = 6.6192(6), *b* = 19.7039(17), *c* = 14.2723(13) Å, *α* = 90°, *β* = 99.079(5)°, *γ* = 90°, *V* = 1841.78(14) Å^3^, *Z* = 4, density (calculated) = 1.389 g m^−3^, *μ* = 0.866 mm^−1^, *F*(000) = 808.0, reflection collected/unique = 10 609/2973, refinement method = full-matrix least-squares on *F*^2^, Final *R* indices [*I* > 2sigma(*I*)]: *R*_1_ = 0.0461, w*R*_2_ = 0.1239, *R* indices (all data): *R*_1_ = 0.0507, w*R*_2_ = 0.1332, goodness of fit on *F*^2^ = 1.074. CCDC 1984253 (Fig. 9B).

##### 4.1.3.3. (2*E*,4*E*)-5-(Benzo[*d*][1,3]dioxol-5-yl)-*N*′-((*E*,*Z*)-4-hydroxy-3-methoxybenzylidene)penta-2,4-dienehydrazide (5c)

Pale yellow solid, m.p. 210–213 °C, yield: 76.4%; IR: 3401, 3231, 3029, 2909, 2831, 1624, 1606, 1507, 1445, 1375, 1337, 1258. ^1^H NMR (600 MHz, DMSO-*d*_6_) *δ* 11.44 (s, 0.55H, NH), 11.25 (s, 0.43H, NH), 9.54 (s, 0.53H, OH), 9.50 (s, 0.39H, OH), 8.13 (s, 0.51H, 1H, CH=N), 7.93 (s, 0.40H, 1H, CH=N), 7.50–7.23 (m, 3H), 7.19–7.06 (m, 2H), 7.03 (dd, *J* = 10.3, 7.8 Hz, 1H), 6.99 (s, 1H), 6.93 (d, *J* = 13.2 Hz, 1H), 6.85 (s, 1H), 6.18 (d, *J* = 14.9 Hz, 1H), 6.06 (d, *J* = 1.7 Hz, 2H, –OCH_2_O–), 3.85 (d, *J* = 18.9 Hz, 3H, OCH_3_). ^13^C NMR (150 MHz, DMSO-*d6*) *δ* 166.46, 161.95, 149.39, 149.11, 148.48, 148.44, 148.37, 147.38, 143.74, 142.86, 141.50, 139.63, 139.35, 131.25, 126.26, 126.22, 125.68, 123.41, 123.01, 122.51, 121.41, 120.34, 116.06, 115.89, 110.25, 109.47, 108.94, 106.16, 101.79, 56.18. C_20_H_18_N_2_O_5_, crystal dimensions 0.12 × 0.1 × 0.1 mm^3^, *M*_r_ = 366.36, monoclinic, space group *P*2_1_/*c* (14) cell: *a* = 18.1988(7), *b* = 4.8105(2), *c* = 22.1672(8) Å, *α* = 90°, *β* = 112.874(3)°, *γ* = 90°, *V* = 1788.03(13) Å^3^, *Z* = 4, density (calculated) = 1.361 g m^−3^, *μ* = 0.822 mm^−1^, *F*(000) = 768.0, reflection collected/unique = 14 927/3069, refinement method = full-matrix least-squares on *F*^2^, final *R* indices [*I* > 2sigma(*I*)]: *R*_1_ = 0.0490, w*R*_2_ = 0.1431, *R* indices (all data): *R*_1_ = 0.0687, w*R*_2_ = 0.1575, goodness of fit on *F*^2^ = 1.052. CCDC 2009516.

##### 4.1.3.4. (2*E*,4*E*)-5-(Benzo[*d*][1,3]dioxol-5-yl)-*N*′-((*E*/*Z*)-4-(dimethylamino)-benzylidene)penta-2,4-dienehydrazide (5d)

Pale yellow solid, m.p. 203–207 °C, yield: 69.1%; IR: 3136, 3053, 2932, 2853, 1640, 1610, 1497, 1447, 1372, 1340, 1260. ^1^H NMR (600 MHz, DMSO-*d6*) *δ* 11.31 (s, 0.54H, NH), 11.12 (s, 0.42H, NH), 8.08 (s, 0.56H, CH), 7.93 (s, 0.44H, CH), 7.52 (dd, *J* = 8.9, 2.7 Hz, 2H), 7.43–7.24 (m, 2H), 7.22–6.89 (m, 4H), 6.74 (dd, *J* = 8.8, 4.2 Hz, 2H), 6.15 (d, *J* = 14.9 Hz, 1H), 6.06 (d, *J* = 2.4 Hz, 2H, –OCH_2_O–), 2.97 (s, 6H, N(CH_3_)_2_). ^13^C NMR (150 MHz, DMSO-*d6*) *δ* 166.26, 151.92, 151.75, 148.44, 148.39, 148.33, 147.63, 141.22, 131.33, 131.29, 128.93, 128.79, 128.52, 128.38, 123.42, 122.91, 122.14, 112.39, 112.13, 109.02, 108.87, 106.21, 106.06, 101.78, 40.40. C_21_H_24.75_N_3_O_4.42_, crystal dimensions 0.12 × 0.04 × 0.03 mm^3^, *M*_r_ = 389.87, monoclinic, space group *P*2_1_/*n* (14) cell: *a* = 13.6957(16), *b* = 6.3687(7), *c* = 24.058(3) Å, *α* = 90°, *β* = 104.090(6)°, *γ* = 90°, *V*=2035.3(4) Å^3^, *Z* = 4, density (calculated) = 1.272 g m^−3^, *μ* = 0.740 mm^−1^, *F*(000) = 828.0, reflection collected/unique = 9153/2692, refinement method = full-matrix least-squares on *F*^2^, final *R* indices [*I* > 2sigma(*I*)]: *R*_1_ = 0.0706, w*R*_2_ = 0.1880, *R* indices (all data): *R*_1_ = 0.1081, w*R*_2_ = 0.2150, goodness of fit on *F*^*2*^ = 1.029. CCDC 1984254.

##### 4.1.3.5. (2*E*,4*E*)-5-(Benzo[*d*][1,3]dioxol-5-yl)-*N*′-((*E*)-4-(diethylamino)-2-hydroxy benzylidene)penta-2,4-dienehydrazide (5e)

The spectroscopic analysis matched with those previously our work published^35^ and included in appendix A.

##### 4.1.3.6. (2*E*,4*E*)-5-(Benzo[*d*][1,3]dioxol-5-yl)-*N*′-(bis(4-fluorophenyl)methylene)penta-2,4-dienehydrazide (5f)

Deep yellow solid, m.p. 190–193 °C, yield: 71.7%; IR: 3276, 3052, 2989, 2895, 1650, 1623, 1595, 1486, 1384, 1339, 1255. ^1^H NMR (600 MHz, DMSO-*d6*) *δ* 10.24 (s, 0.59H, NH), 9.61 (s, 0.44H, NH), 7.52 (d, *J* = 2.9 Hz, 2H), 7.41 (s, 2H), 7.38 (d, *J* = 8.4 Hz, 2H), 7.31 (s, 1H), 7.24 (s, 3H), 6.92 (t, *J* = 4.6 Hz, 4H), 6.36 (d, *J* = 14.7 Hz, 1H), 6.05 (s, 2H, –OCH_2_O-). ^13^C NMR (151 MHz, DMSO-*d6*) *δ* 164.02, 162.39, 148.44, 143.81, 141.82, 139.57, 131.69, 131.17, 129.97, 126.24, 125.51, 123.41, 117.00, 115.92, 108.98, 106.16, 101.81. ^19^F NMR (565 MHz, DMSO-*d6*) *δ* −106.46. C_25_H_18_F_2_N_2_O_3_, crystal dimensions 0.20 × 0.12 × 0.10 mm^3^, *M*_r_ = 432.41, monoclinic, space group *P*2_1_/*n* (14) cell: *a* = 5.329(3), *b* = 10.708(5), *c* = 15.496(7)Å, *α* = 83.878(9)°, *β* = 87.608(9)°, *γ* = 82.240(9)°, *V*=870.8(7) Å^3^, *Z* = 4, density (calculated) = 1.649 g m^−3^, *F*(000) = 448, reflection collected/unique = 6085/3045, refinement method = full-matrix least-squares on *F*^2^, final *R* indices [*I* > 2sigma(*I*)]: *R*_1_ = 0.0623, w*R*_2_ = 0.1690, *R* indices (all data): *R*_1_ = 0.0965, w*R*_2_ = 0.1941, goodness of fit on *F*^*2*^ = 1.029. CCDC 1984255.

##### 4.1.3.7. (2*E*,4*E*)-5-(Benzo[*d*][1,3]dioxol-5-yl)-*N*′-((*E*)-3,5-di-*tert*-butyl-2-hydroxy-benzylidene)penta-2,4-dienehydrazide (5g)

Deep yellow solid, m.p. 208–211 °C, yield: 72.3%; IR: 3449, 3360, 3050, 2957, 2820, 1663, 1615, 1594, 1445, 1385, 1362, 1254. ^1^H NMR (600 MHz, DMSO-*d6*) *δ* 12.19 (s, 1H, NH), 11.94 (s, 1H, OH), 8.36 (s, 1H, CH=N), 7.40 (dd, *J* = 15.0, 9.7 Hz, 1H), 7.30 (s, 2H), 7.24 (d, *J* = 2.3 Hz, 1H), 7.03 (dd, *J* = 12.9, 3.2 Hz, 3H), 6.94 (s, 1H), 6.19 (d, *J* = 14.9 Hz, 1H), 6.06 (s, 2H, –OCH_2_O-), 1.41 (s, 9H, 3CH_3_), 1.26 (s, 9H, 3CH_3_). ^13^C NMR (151 MHz, DMSO-*d6*) *δ* 161.93, 155.08, 150.34, 148.51, 148.46, 142.49, 140.88, 140.08, 136.04, 131.15, 126.20, 125.94, 125.55, 123.60, 121.85, 117.55, 108.98, 106.20, 101.82, 35.11, 34.36, 31.77, 29.76.

##### 4.1.3.8. (2*E*,4*E*)-5-(Benzo[*d*][1,3]dioxol-5-yl)-*N*′-((*E*)-1-(4-bromophenyl)ethylidene)penta-2,4-dienehydrazide (5h)

Deep yellow solid, m.p. 188–192 °C, yield: 69.1%; IR: 3285, 3061, 2941, 2811, 2779, 1644, 1607, 1539, 1497, 1447, 1370, 1256. ^1^H NMR (600 MHz, DMSO-*d6*) *δ* 10.60 (s, 1H, OH), 7.77 (d, *J* = 7.6 Hz, 2H), 7.62 (d, *J* = 8.5 Hz, 2H), 7.38 (dd, *J* = 27.5, 13.6 Hz, 2H), 7.14 (d, *J* = 14.1 Hz, 1H), 7.01 (dd, *J* = 29.7, 6.8 Hz, 2H), 6.93 (d, *J* = 8.0 Hz, 1H), 6.48 (d, *J* = 14.9 Hz, 1H), 6.06 (s, 2H, –OCH_2_O-), 2.28 (d, *J* = 16.7 Hz, 3H). ^13^C NMR (151 MHz, DMSO-*d6*) *δ* 166.56, 148.46, 146.69, 143.03, 141.61, 139.65, 139.41, 131.24, 129.11, 128.73, 127.39, 126.26, 125.68, 123.44, 122.94, 120.27, 114.77, 108.95, 106.16, 101.79, 28.41.

### 4.2. Biology

#### 4.2.1. Screening of cytotoxic activity

The methodology of the NCI anticancer screening has been described in detail elsewhere (https://dtp.cancer.gov/).^52^

#### 4.2.2. Sirtuin inhibitory assay

Sirt-1 and Sirt-2 inhibitory assay was performed to evaluate the inhibitory potency of 5a–h against Sirt-1 and Sirt-2.^53^ See ESI.†

##### 4.2.2.1. Cell apoptosis assay

Apoptosis was determined by flow cytometry based on the Annexin-V-fluoresce in isothiocyanate (FITC) and propidium iodide (PI) staining kit (BD Pharmingen, San Diego, USA).^54,55^ See ESI.†

## Conflicts of interest

The authors declare no competing financial interest.

## Supplementary Material

RA-011-D1RA04061H-s001

RA-011-D1RA04061H-s002

RA-011-D1RA04061H-s003
