# Supplementary material for: Structure-based design, synthesis, and biological evaluation of novel piperine–resveratrol hybrids as antiproliferative agents targeting SIRT-2
Source: RSC Adv. 2021 Jul 27;11(41):25738–51. doi: 10.1039/d1ra04061h (PMC9037111; doi:10.1039/d1ra04061h)
Supplement: RA-011-D1RA04061H-s001 [file RA-011-D1RA04061H-s001.pdf]

Electronic Supplementary Material (ESI) for RSC Advances.
This journal is © The Royal Society of Chemistry 2021

Appendix A

Experimental

General details

Solvents and chemical reagents were of reagent grade and were used as received. Piperine and hydrazine (≥99.0%) were obtained from Aladdin Industrial Corporation (Shanghai, China). Oxalyl chloride (≥99.0%) was obtained from TCI (Tokyo Chemical Industry Co. Ltd). 4-(Diethylamino)salicylaldehyde, 2,4-Dihydroxybenzaldehyde, 4-hydroxybenzaldehyde were purchased from Alfa Aesar China Co. Ltd. (Shanghai, China). 4-Hydroxy-3-methoxybenzaldehyde, 4-(Dimethylamino)benzaldehyde, 4-bromoacetophenone and 4,4′-Difluorobenzophenone were obtained from Aladdin Industrial Corporation (Shanghai, China).Proton (^{1}H NMR), carbon (^{13}C NMR) and flour (^{19}F NMR) nuclear magnetic resonance spectra were recorded in DMSO-d^6 or $CDCl_3$ on a Bruker Avance 600 MHz instrument using tetramethylsilane (TMS) as the internal standard. Coupling constants (J) were reported in Hz and referred to apparent peak multiplications. Melting points of the compounds were determined on a XT-4 binocular microscope (Tianjin Analytical Instrument Factory, China) and were not corrected. X-ray data were collected using a BRUKER SMART APEX-CCD diffractometer with Cu-Kα radiation (λ = 0.71073 Å). The infrared spectra of the generated compounds were done on FT-IR spectra recorded in KBr on a thermo nicolet iS10 FTIR spectrophotometer.

Synthesis of (2*E*,4*E*)-5-(benzo[d][1,3]dioxol-5-yl)penta-2,4-dienoyl chloride (3)

The experimental procedure was carried out according to our previous work [1]. Oxalyl chloride (1.24 g, 10 mmol) in DCM (5 mL) and three drops of DMF as a catalyst were added to piperic acid (217 mg, 1.01 mmol) and stirred at room temperature for 4 h. After completing the reaction, the oxalyl chloride excess and solvent were then evaporated. The obtained acid chloride was dissolved in DCM and taken directly to the next step.

Spectroscopic analysis and X-ray single crystal for compound 5e.

(2E,4E)-5-(benzo[d][1,3]dioxol-5-yl)-N'-((E)-4-(diethylamino)-2-hydroxy benzylidene)penta-2,4-dienehydrazide (5e): Yellow solid, m.p. 105-107°C; IR: 3208, 3034, 2972, 2884, 1629, 1547, 1518, 1443, 1371, 1246. ^{1}H NMR (600 MHz, DMSO-d_6) δ 11.52 (s, 1H), 11.34 (s, 1H), 8.20 (s, 1H), 7.36 – 7.28 (m, 2H), 7.20 (d, J = 11.6 Hz, 1H), 7.02 (d, J = 14.7 Hz, 2H), 6.98 – 6.90 (m, 2H), 6.13 (d, J = 14.9 Hz, 1H), 6.11 (d, J = 2.3 Hz, 1H), 6.06 (s, 2H), 3.35 (s, 4H), 1.11 (s, 6H). ^{13}C NMR (151 MHz, DMSO-d_6) δ 161.26, 160.10, 150.52, 148.91, 148.34, 141.39, 139.36, 131.92, 131.69, 125.64, 123.41, 122.84, 122.23, 108.95, 107.01, 106.21, 106.21, 103.80, 101.78, 44.36, 12.86.

Crystallographic description of ***5-(2H-1,3-benzodioxol-5-yl)-N'-((4-(diethylamino)-2-hydroxyphenyl)methylidene)penta-2,4-dienehydrazide methanol solvate*** **(5e):** $C_{23}H_{25}N_3O_4.CH_3OH$, crystal dimensions 0.15 x 0.15 x 0.1 mm^3, M_r = 439.50, monoclinic, space group P 2_1/c (14), Cell: a = 11.298(3), b = 13.398(3), c = 15.678(4) Å, α = 90°, β = 107.247(4)°, γ = 90°, V = 2266.5(10) Å^3, Z = 4, Density (calculated) = 1.288 Mg/m^3, μ = 0.091 mm^{-1}, F(000)= 936, reflection collected / unique = 17545 / 4630, refinement method = full-matrix least-squares on F2, Final R indices [I>2sigma(I)]: $R1$ = 0.0475, $wR2$ = 0.1263, R indices (all data): $R1$ = 0.0948, $wR2$ =

0.1535, goodness of fit on F^2 = 1.037. CCDC 1934954 for ***5-(2H-1,3-benzodioxol-5-yl)-N'-((4-(diethylamino)-2-hydroxyphenyl)methylidene)penta-2,4-dienehydrazide methanol solvate*** **(5e):** contains the supplementary crystallographic data for this paper. These data can be obtained free of charge from The Cambridge Crystallographic Data Centre via www.ccdc.cam.ac.uk/data_request/cif.

Evaluation of *in vitro* antiproliferative activity for compounds 5a-h

Compounds **5a-h** were selected by National Cancer Institute (NCI) for *in vitro* anticancer screening. The methodology of the NCI anticancer screening has been described in details elsewhere (https://dtp.cancer.gov/).

Process

The operation of *in vitro* screen utilizes 60 different human tumor cell lines, representing leukemia, melanoma and cancers of the lung, colon, brain, ovary, breast, prostate, and kidney.

The screening is a two-stage process, beginning with the evaluation of all compounds against the 60 cell lines at a single dose of 10^{-5} molar (10 uM). Compounds which exhibit significant growth inhibition are again evaluated against the 60 cell lines panel at five concentration levels, 10-fold dilutions each with the top dose being 10^{-4} molar (100 uM).

Methodology of the *in vitro* anticancer screening

The human tumor cell lines of the cancer screening panel are grown in Roswell Park Memorial Institute (RPMI) 1640 medium containing 5% fetal bovine serum and 2 mM L-glutamine. For a typical screening experiment cells are inoculated into 96 well microtiter plates in 100 µL at plating densities ranging from 5,000 to 40,000 cells/well depending on the doubling time of individual cell lines. After cell inoculation, the

microtiter plates are incubated at 37 C, 5% CO2, 95% air and 100% relative humidity for 24 h prior to addition of experimental drugs.

After 24 h, two plates of each cell line are fixed *in situ* with TCA, to represent a measurement of the cell population for each cell line at the time of drug addition (Tz). Experimental drugs are solubilized in dimethyl sulfoxide at 400-fold the desired final maximum test concentration and stored frozen prior to use. At the time of drug addition, an aliquot of frozen concentrate is thawed and diluted to twice the desired final maximum test concentration with complete medium containing 50 μg/mL gentamicin. Additional four, 10-fold or ½ log serial dilutions are made to provide a total of five drug concentrations plus control. Aliquots of 100 μL of these different drug dilutions are added to the appropriate microtiter wells already containing 100 μL of medium, resulting in the required final drug concentrations.

Following drug addition, the plates are incubated for an additional 48 h at 37 C, 5% CO_2, 95% air, and 100% relative humidity. For adherent cells, the assay is terminated by the addition of cold TCA. Cells are fixed *in situ* by the gentle addition of 50 μL of cold 50% (w/v) TCA (final concentration, 10% TCA) and incubated for 60 minutes at 4 C. The supernatant is discarded, and the plates are washed five times with tap water and air dried. Sulforhodamine B (SRB) solution (100 μL) at 0.4% (w/v) in 1% acetic acid is added to each well, and plates are incubated for 10 minutes at room temperature. After staining, unbound dye is removed by washing five times with 1% acetic acid and the plates are air dried. Bound stain is subsequently solubilized with 10 mM trizma base, and the absorbance is read on an automated plate reader at a wavelength of 515 nm. For suspension cells, the methodology is the same except that the assay is terminated by fixing settled cells at the bottom of the wells by gently adding 50 μL of 80% TCA (final concentration, 16% TCA).

Using the seven absorbance measurements [time zero (Tz), control growth (C), and test growth in the presence of drug at the five concentration levels (Ti)], the percentage growth is calculated at each of the drug concentrations levels. Percentage growth inhibition is calculated as:

[(Ti-Tz)/(C-Tz)] x 100 for concentrations for which Ti >/= Tz

[(Ti-Tz)/Tz] x 100 for concentrations for which Ti < Tz

Three dose response parameters are calculated for each experimental agent. Growth inhibition of 50% (GI50) is calculated from [(Ti-Tz)/(C-Tz)] x 100 = 50, which is the drug concentration resulting in a 50% reduction in the net protein increase (as measured by SRB staining) in control cells during the drug incubation. The drug concentration resulting in total growth inhibition (TGI) is calculated from Ti = Tz. The LC_{50} (concentration of drug resulting in a 50% reduction in the measured protein at the end of the drug treatment as compared to that at the beginning) indicating a net loss of cells following treatment is calculated from [(Ti-Tz)/Tz] x 100 = -50. Values are calculated for each of these three parameters if the level of activity is reached; however, if the effect is not reached or is exceeded, the value for that parameter is expressed as greater or less than the maximum or minimum concentration tested.

Cell cycle analysis and apoptosis assay

Cell cycle analysis

Cell cycle analysis was performed for compound **5b** on MCF-7 cell line. After treatment, the cells were suspended in 0.5 mL of PBS, collected by centrifugation, and fixed in ice-cold 70% (v/v) ethanol washed with PBS, resuspended with 0.1 mg/mL RNase, stained with 40 mg/ml PI, and analyzed by flow cytometry using FACScalibur (Becton Dickinson). The cell cycle distributions were calculated using Phoenix Flow Systems and Verity Software House

Apoptosis assay

The MCF-7 cell line was treated with IC_{50} of compound **5b** for 24 h. After treatment, the cells were suspended in 0.5 mL of PBS, collected by centrifugation, Resuspend cells in 500 µl of 1X Binding Buffer. Add 5 µl of Annexin V-FITC and 5 µl of propidium iodide (PI 50mg/ml, optional.) Incubate at room temperature for 5 min in the dark, suspended in 5 mL PBS and centrifuged for 5 min, re-suspended with 1 mL PI staining solution (0.1 mg/ml RNase) + PE Annexin V (component no. 51-65875X) and kept in dark at 37 °C for 10 min, finally analyzed by flow cytometry using FACScalibur (Becton Dickinson).

References

[1] A.H. Tantawy, S.M. Farag, L. Hegazy, H. Jiang, M.Q. Wang, The larvicidal activity of natural inspired piperine-based dienehydrazides against Culex pipiens, Bioorg. Chem. (2019) 103464. https://doi.org/10.1016/j.bioorg.2019.103464.
