# Supplementary material for: Structure-based design, synthesis, and biological evaluation of novel piperine–resveratrol hybrids as antiproliferative agents targeting SIRT-2
Source: RSC Adv. 2021 Jul 27;11(41):25738–51. doi: 10.1039/d1ra04061h (PMC9037111; doi:10.1039/d1ra04061h)
Supplement: RA-011-D1RA04061H-s002 [file RA-011-D1RA04061H-s002.pdf]

Electronic Supplementary Material (ESI) for RSC Advances.
This journal is © The Royal Society of Chemistry 2021

Structure-based Design, Synthesis, and Biological Evaluation of Novel Piperine-Resveratrol Hybrids as Antiproliferative Agents Targeting SIRT-2

Ahmed H. Tantawy[1,2,3]*, Xiang-Gao Meng[4]*, Adel A. Marzouk[5], Ali Fouad[5], Ahmed H. Abdelazeem[6,7], Bahaa G. M. Youssif[8], Hong Jiang[2]*, Man-Qun Wang[1]*

[1]Hubei Insect Resources Utilization and Sustainable Pest Management Key Laboratory, College of Plant Science and Technology, Huazhong Agricultural University, Wuhan 430070, People's Republic of China, [2]Department of Chemistry, College of Science, Huazhong Agricultural University, Wuhan, 430070, China, [3]Department of Chemistry, College of Science, Benha University, Benha 13518, Egypt; [4]Key Laboratory of Pesticide and Chemical Biology, Ministry of Education, and School of Chemistry, Central China Normal University, Wuhan 430079, China; [5]Department of Pharmaceutical Chemistry, Faculty of Pharmacy, Al-Azhar University, Assiut Branch, Assiut-71524, Egypt; [6]Department of Medicinal Chemistry, Faculty of pharmacy, Beni-Suef University, Beni-Suef 62514, Egypt; [7]Department of Pharmaceutical Sciences, College of Pharmacy, Riyadh Elm University, Riyadh 11681, Saudi Arabia; [8]Pharmaceutical Organic Chemistry Department, Faculty of Pharmacy, Assiut University, Assiut 71526, Egypt.

Supporting information

Table of Contents

IR, ^{1}H and ^{13}C NMR spectra of compound 5a	**Figure S1**
^{1}H and ^{13}C NMR spectra of compound 5b	**Figure S2**
IR, ^{1}H and ^{13}C NMR spectra of compound 5c	**Figure S3**
IR, ^{1}H and ^{13}C NMR spectra of compound 5d	**Figure S4**
IR, ^{1}H and ^{13}C NMR spectra of compound 5e (reported)	**Figure S5**
IR, ^{1}H and ^{13}C NMR spectra of compound 5f	**Figure S6**
IR, ^{1}H and ^{13}C NMR spectra of compound 5g	**Figure S7**
IR, ^{1}H and ^{13}C NMR spectra of compound 5h	**Figure S8**
➢ Hirschfield surfaces in **5a** mapped with (a) d_{norm}; (b) Shape index, (c) full fingerprint plot; (d) H···O contacts (O-H···O and C-H…O); (e) H···C contacts (C-H···π) and (f) C···C contacts (π···π).	**Figure S9**
➢ Hirschfield surfaces in **5c** mapped with (a) d_{norm}; (b) Shape index, (c) full fingerprint plot; (d) H···O contacts (O-H···O and C-H…O); (e) H···C contacts (C-H···π) and (f) C···C contacts (π···π).	**Figure S10**
➢ Hirschfield surfaces in **5d** mapped with (a) d_{norm}; (b) Shape index, (c) full fingerprint plot; (d) H···O contacts (O-H···O and C-H…O); (e) H···C contacts (C-H···π) and (f) C···C contacts (π···π).	**Figure S11**
➢ Hirschfield surfaces in **5e** mapped with (a) d_{norm}; (b) Shape index, (c) full fingerprint plot; (d) H···O contacts (O-H···O and C-H…O); (e) H···C contacts (C-H···π) and (f) C···C contacts (π···π).	**Figure S12**
➢ Hirschfield surfaces in **5f** mapped with (a) d_{norm}; (b) Shape index, (c) full fingerprint plot; (d) H···O contacts (O-H···O and C-H…O); (e) H···C contacts (C-H···π) and (f) C···C contacts (π···π).	**Figure S13**
The crystallographic data and structure refinement for **5a-f**	**Table S1**

Figure S1

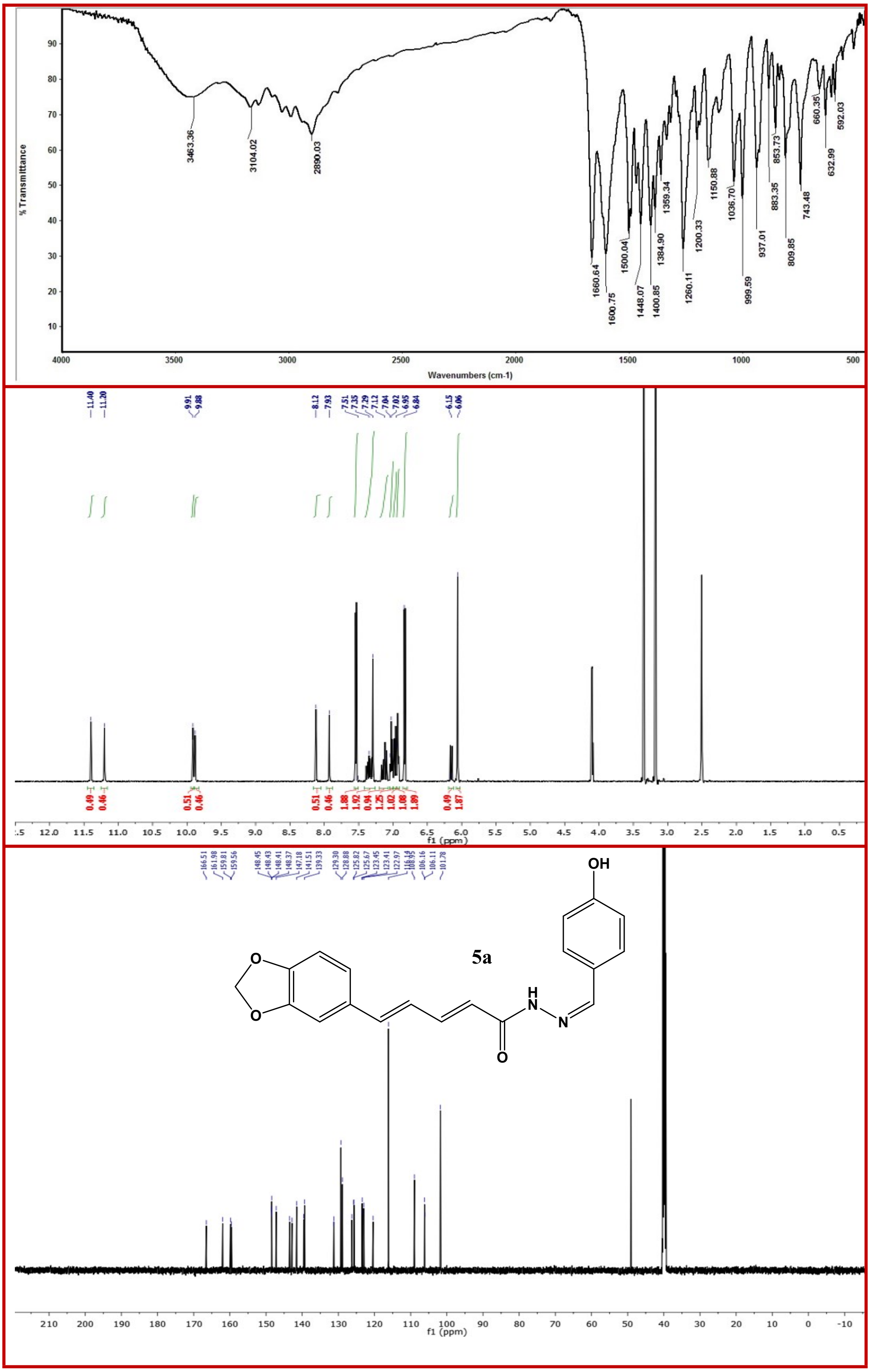

% Transmittance
Wavenumbers (cm-1)
3463.36
3104.02
2890.03
1660.64
1600.75
1500.04
1448.07
1400.85
1384.90
1359.34
1260.11
1200.33
1150.88
1036.70
999.59
937.01
883.35
853.73
809.85
743.48
660.35
632.99
592.03
f1 (ppm)
11.40
11.20
9.91
9.88
8.12
7.93
7.51
7.35
7.29
7.12
7.04
7.02
6.95
6.84
6.15
6.06
0.49
0.46
0.51
0.46
0.51
0.46
1.88
1.92
0.94
1.25
1.02
1.08
1.89
0.49
1.87
166.51
161.98
159.81
159.56
148.45
148.43
148.41
148.37
147.18
141.51
139.33
129.30
128.88
125.82
125.67
123.45
123.41
122.97
106.16
106.11
101.78
5a
OH
H
N
O

Figure S2

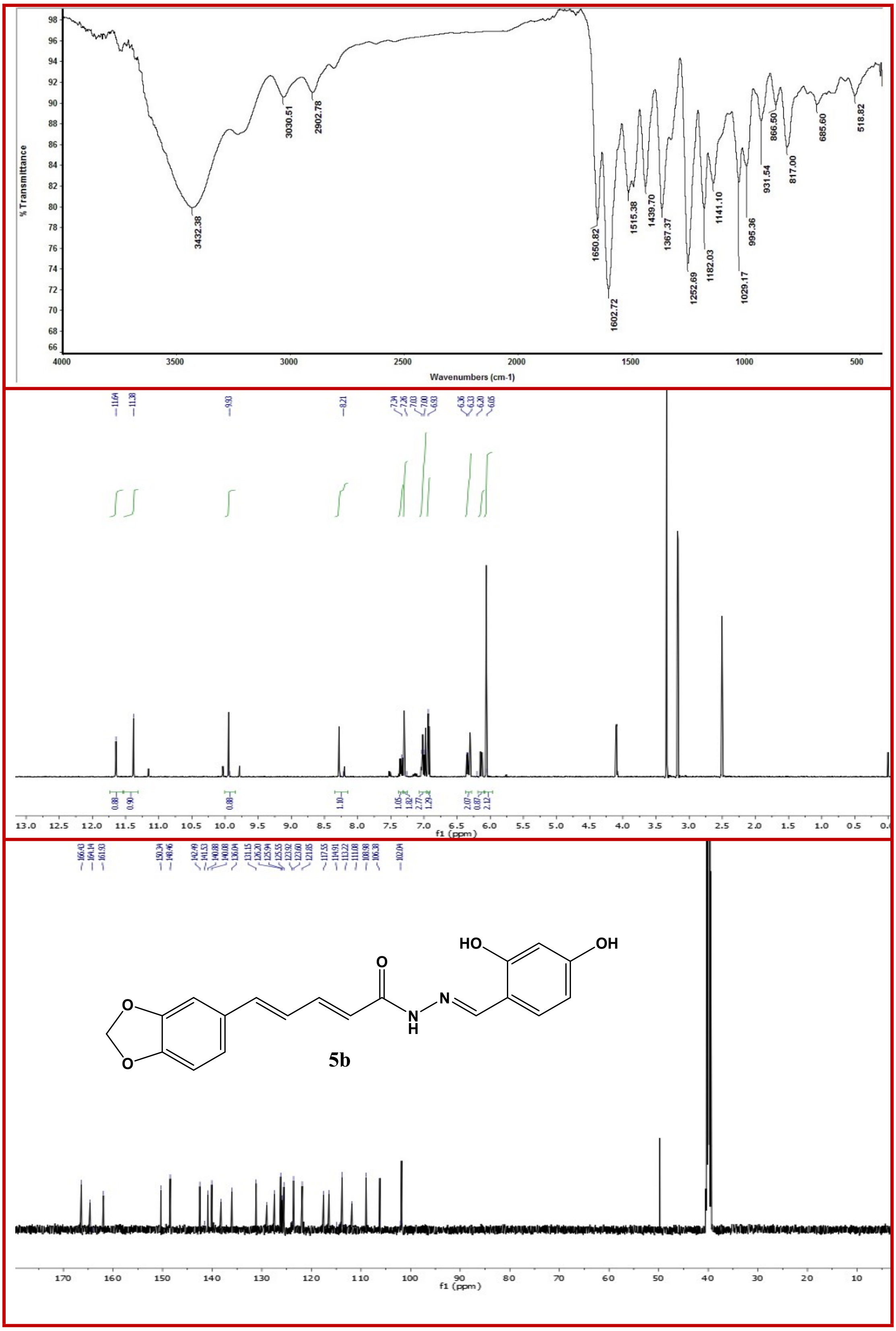

% Transmittance
Wavenumbers (cm-1)
3432.38
3030.51
2902.78
1650.82
1602.72
1515.38
1439.70
1367.37
1252.69
1182.03
1141.10
1029.17
995.36
931.54
866.50
817.00
685.60
518.82
f1 (ppm)
f1 (ppm)
HO
OH
O
N
H
5b

Figure S3

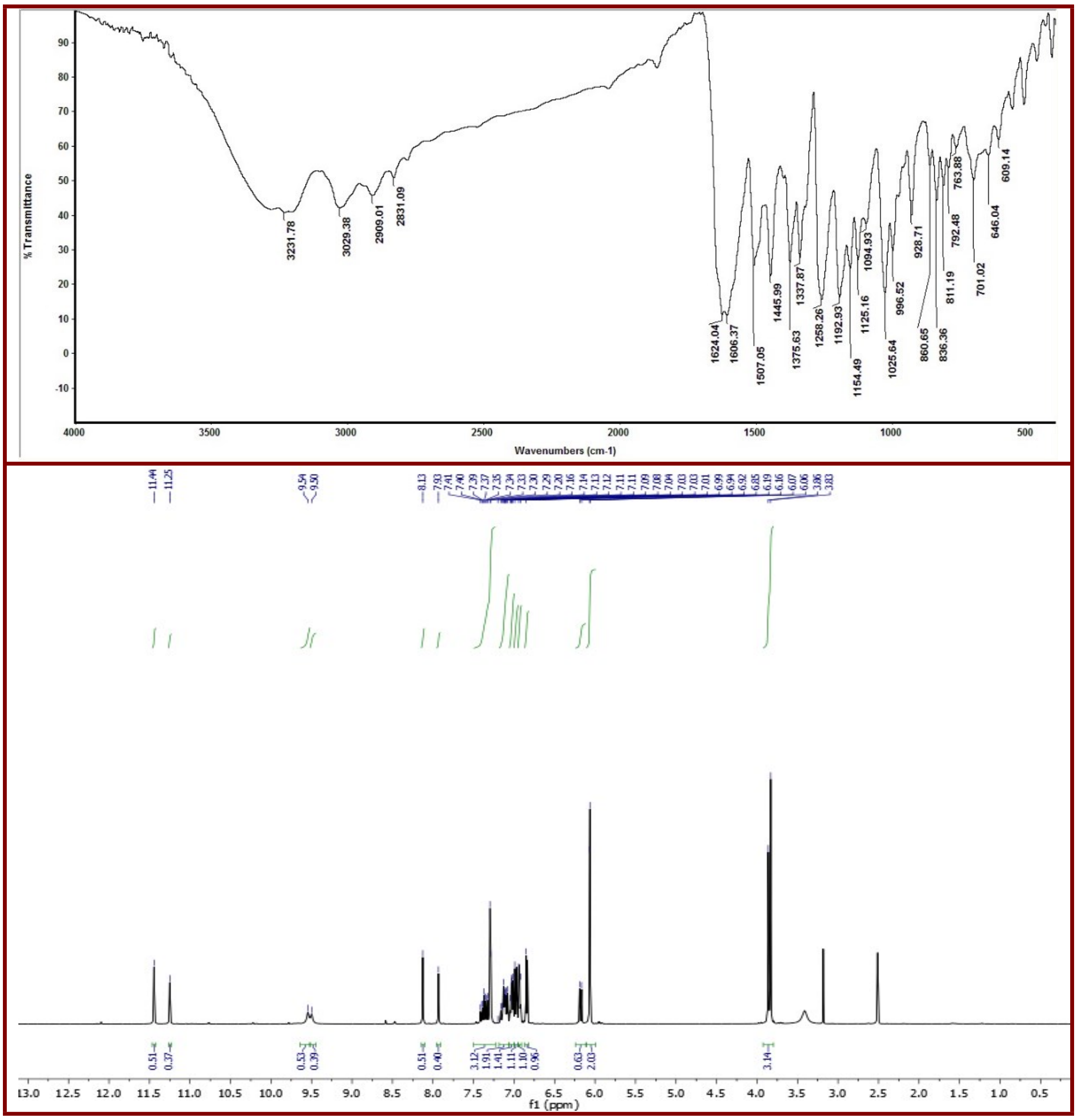
% Transmittance
Wavenumbers (cm-1)
3231.78
3029.38
2909.01
2831.09
1624.04
1606.37
1507.05
1445.99
1375.63
1337.87
1258.26
1192.93
1154.49
1125.16
1094.93
1025.64
996.52
928.71
860.65
836.36
811.19
792.48
763.88
701.02
646.04
609.14
f1 (ppm)
11.44
11.25
9.54
9.50
8.13
7.93
3.86
3.83
0.51
0.37
0.53
0.39
0.51
0.40
3.12
1.91
1.41
1.11
1.10
0.96
0.63
2.03
3.14

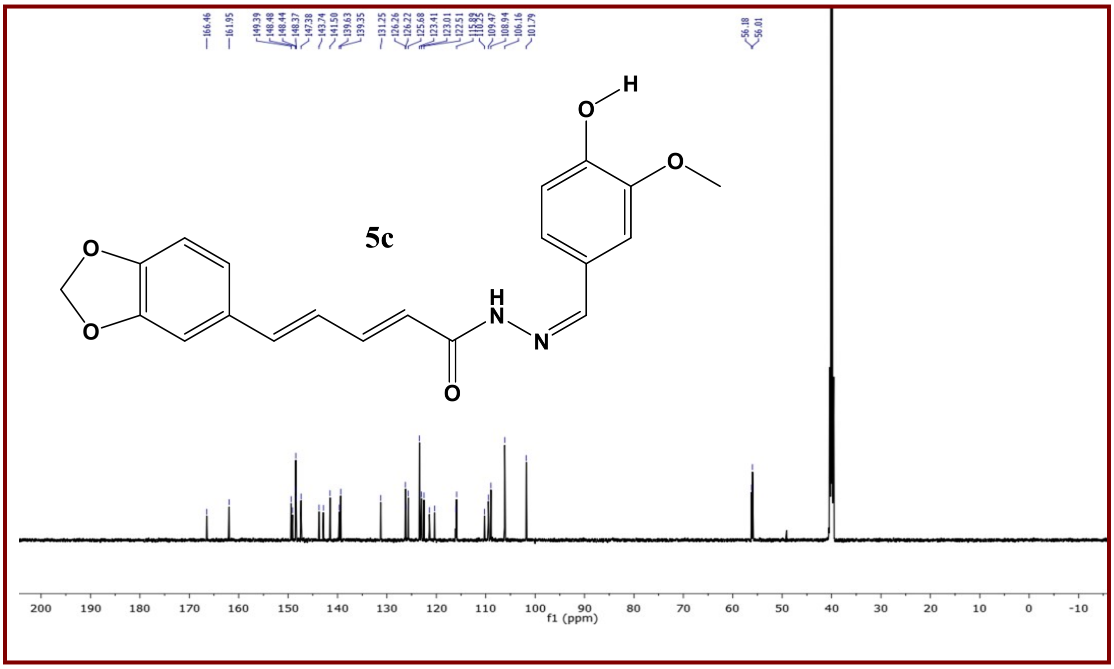

5c
f1 (ppm)

Figure S4

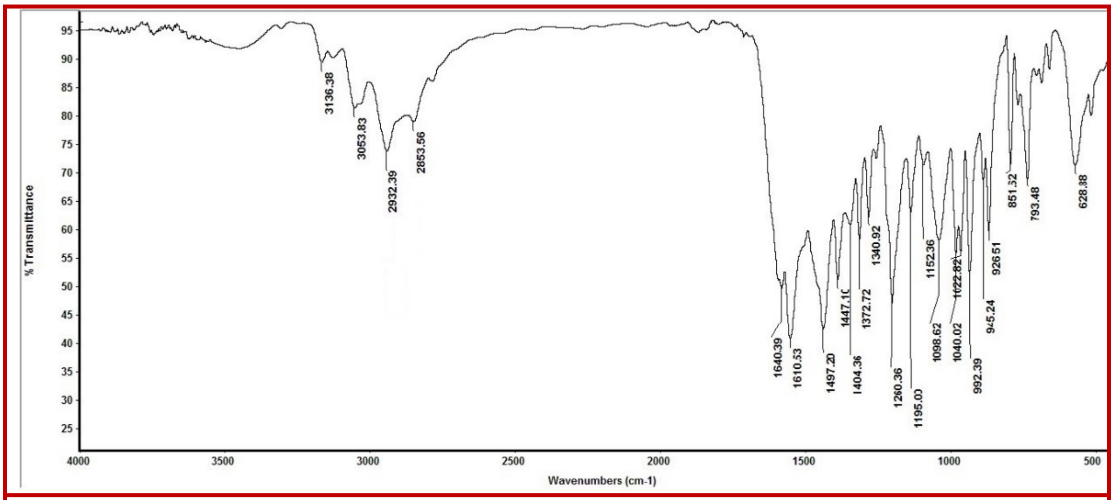

% Transmittance
Wavenumbers (cm-1)
3136.38
3053.83
2932.39
2853.56
1640.39
1610.53
1497.20
1447.10
1404.36
1372.72
1340.92
1260.36
1195.03
1152.36
1098.62
1040.02
992.39
945.24
926.51
851.32
793.48
628.38

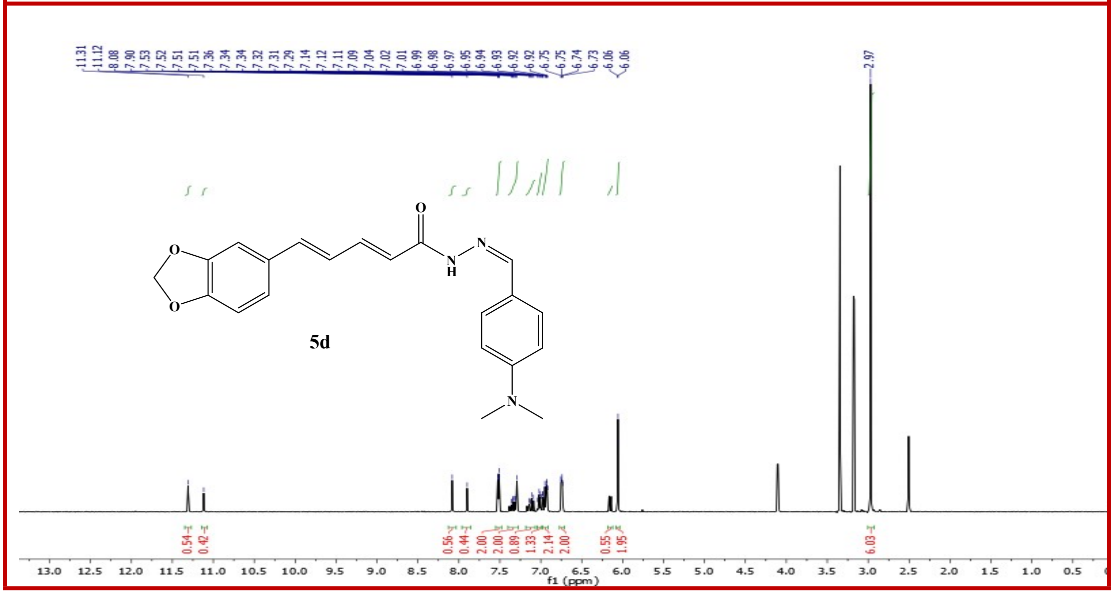

5d
f1 (ppm)

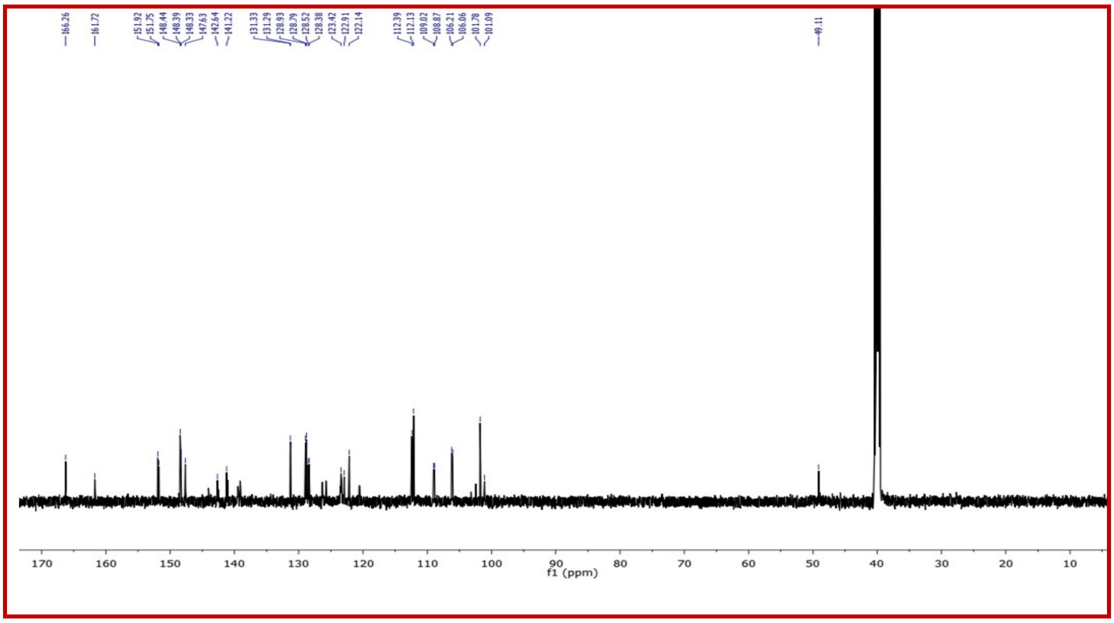
166.26
161.72
151.92
151.75
148.44
148.39
148.33
147.63
142.64
141.22
131.33
131.29
128.93
128.79
128.52
128.38
123.42
122.91
122.14
112.39
112.13
109.02
108.87
106.21
106.06
101.78
101.09
49.11
170 160 150 140 130 120 110 100 90 80 70 60 50 40 30 20 10
f1 (ppm)

Figure S5

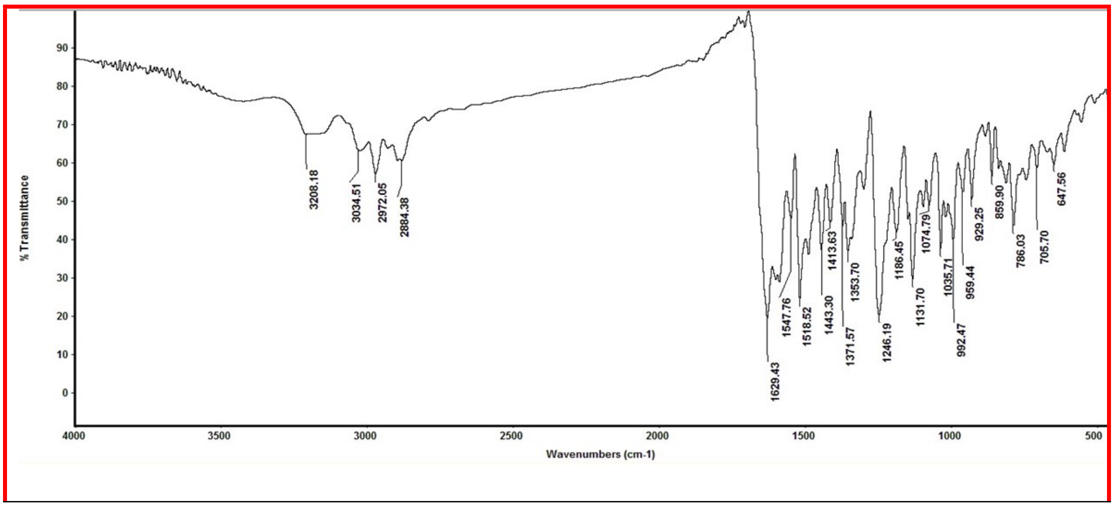
% Transmittance
90 80 70 60 50 40 30 20 10 0
3208.18
3034.51
2972.05
2884.38
1629.43
1547.76
1518.52
1443.30
1413.63
1371.57
1353.70
1246.19
1186.45
1131.70
1074.79
1035.71
992.47
959.44
929.25
859.90
786.03
705.70
647.56
4000 3500 3000 2500 2000 1500 1000 500
Wavenumbers (cm-1)

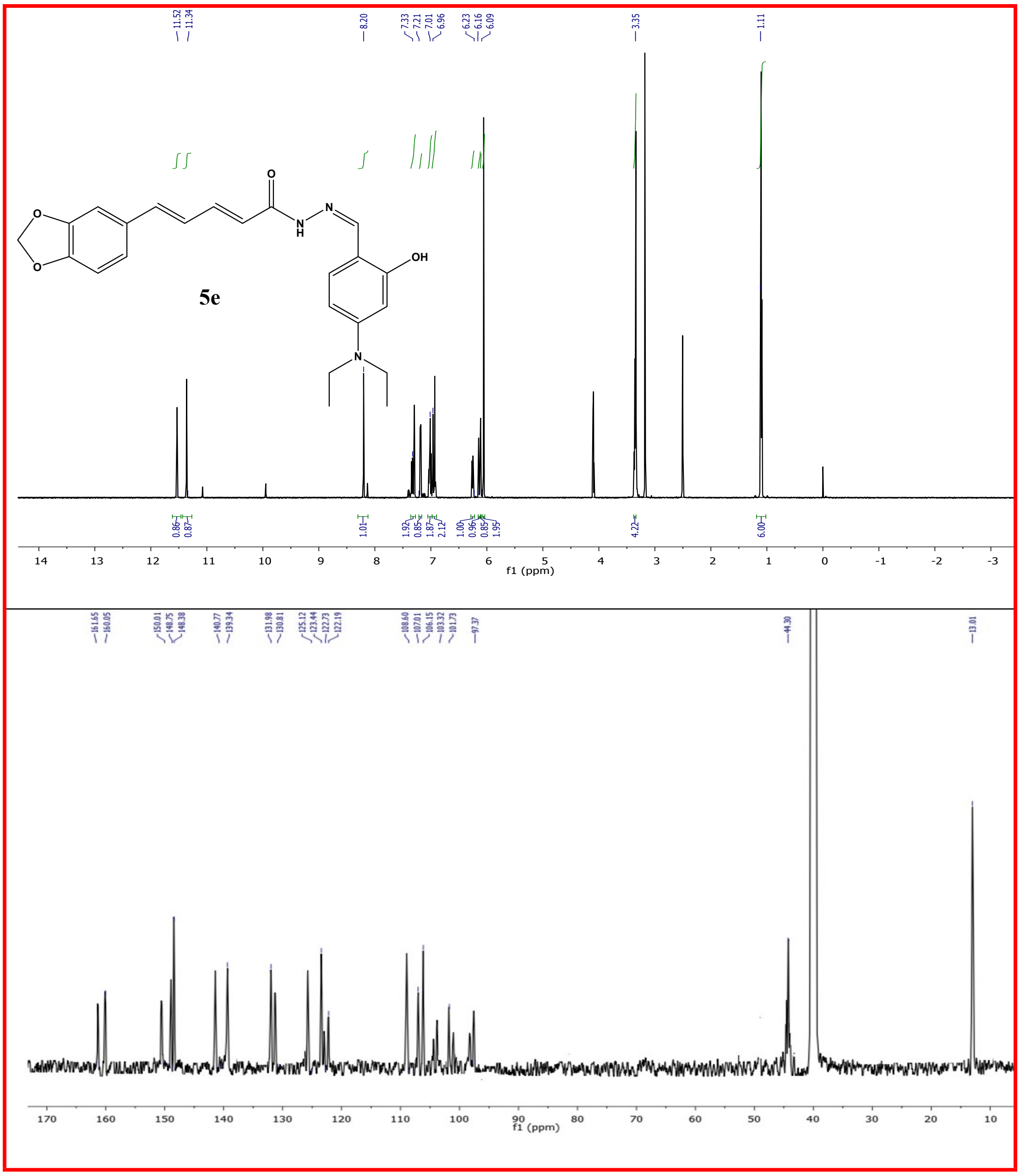
11.52
11.34
8.20
7.33
7.21
7.01
6.96
6.23
6.16
6.09
3.35
1.11
5e
OH
f1 (ppm)
161.65
160.05
150.01
148.75
148.38
140.77
139.34
131.98
130.81
125.12
123.44
122.73
122.19
108.60
107.01
106.15
103.32
101.73
97.37
44.30
13.01
f1 (ppm)

Figure S6

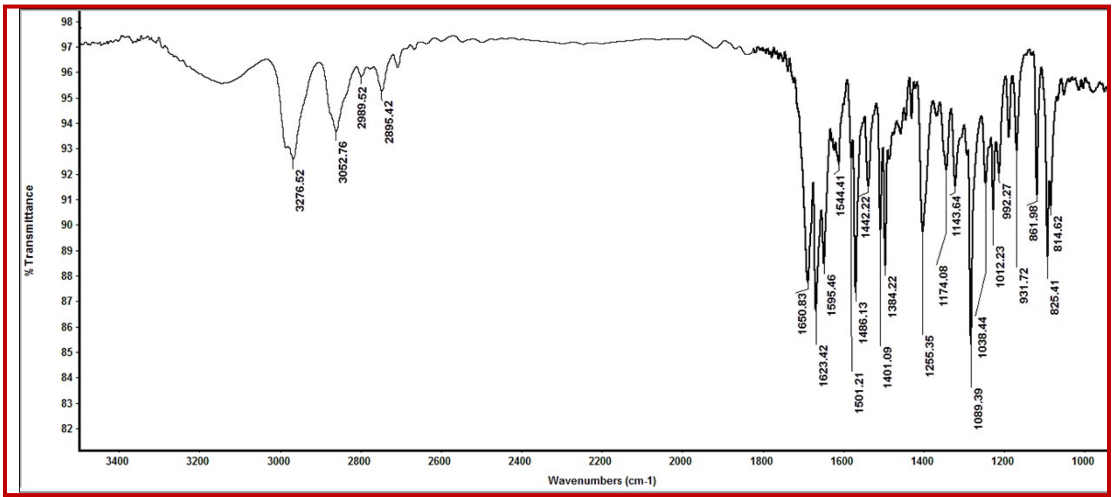

% Transmittance
Wavenumbers (cm-1)
3276.52
3052.76
2989.52
2895.42
1650.83
1623.42
1595.46
1544.41
1501.21
1486.13
1442.22
1401.09
1384.22
1255.35
1174.08
1143.64
1089.39
1038.44
1012.23
992.27
931.72
861.98
825.41
814.62

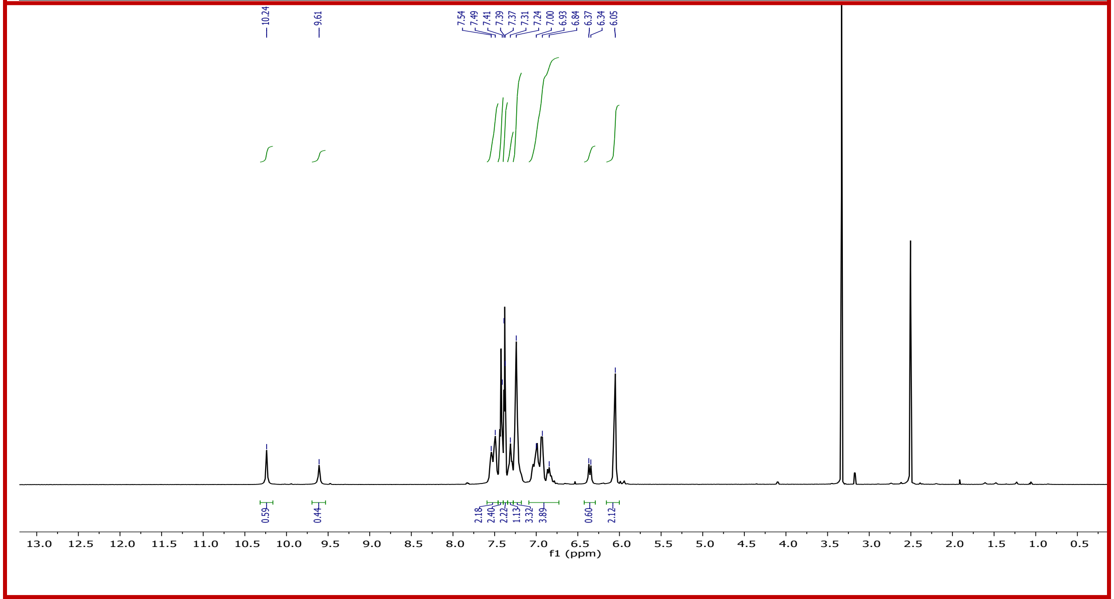

10.24
9.61
7.54
7.49
7.41
7.39
7.37
7.31
7.24
7.00
6.93
6.84
6.37
6.34
6.05
0.59
0.44
2.18
2.40
2.22
1.13
3.32
3.89
0.60
2.12
f1 (ppm)

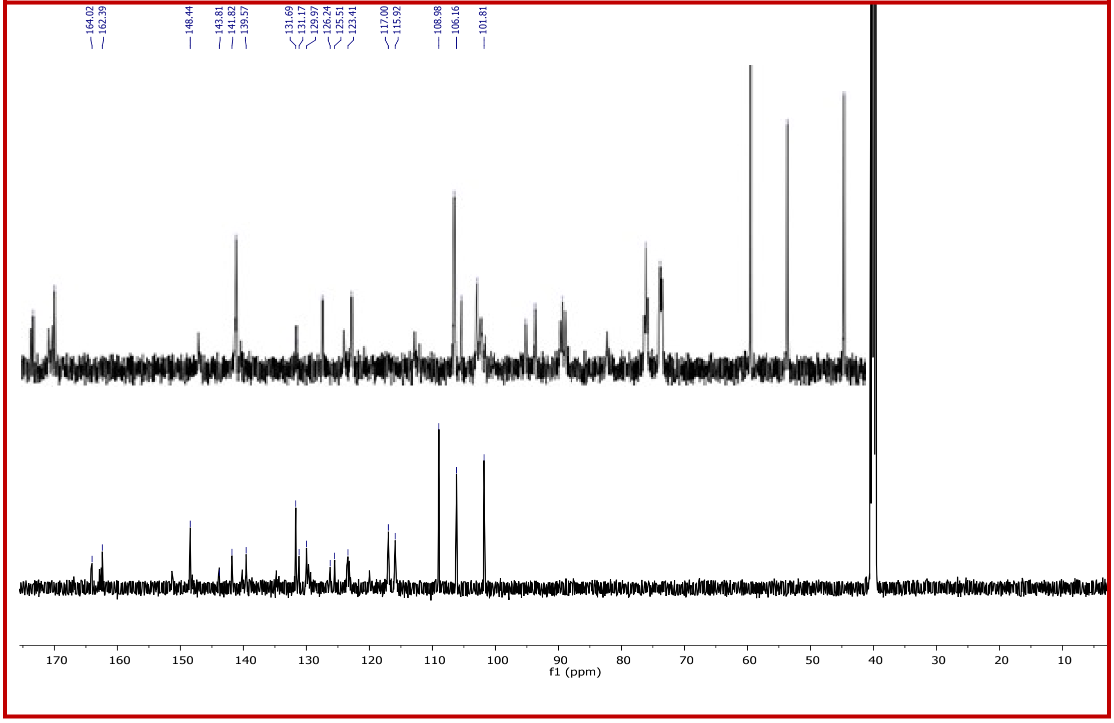

164.02
162.39
148.44
143.81
141.82
139.57
131.69
131.17
129.97
126.24
125.51
123.41
117.00
115.92
108.98
106.16
101.81
f1 (ppm)

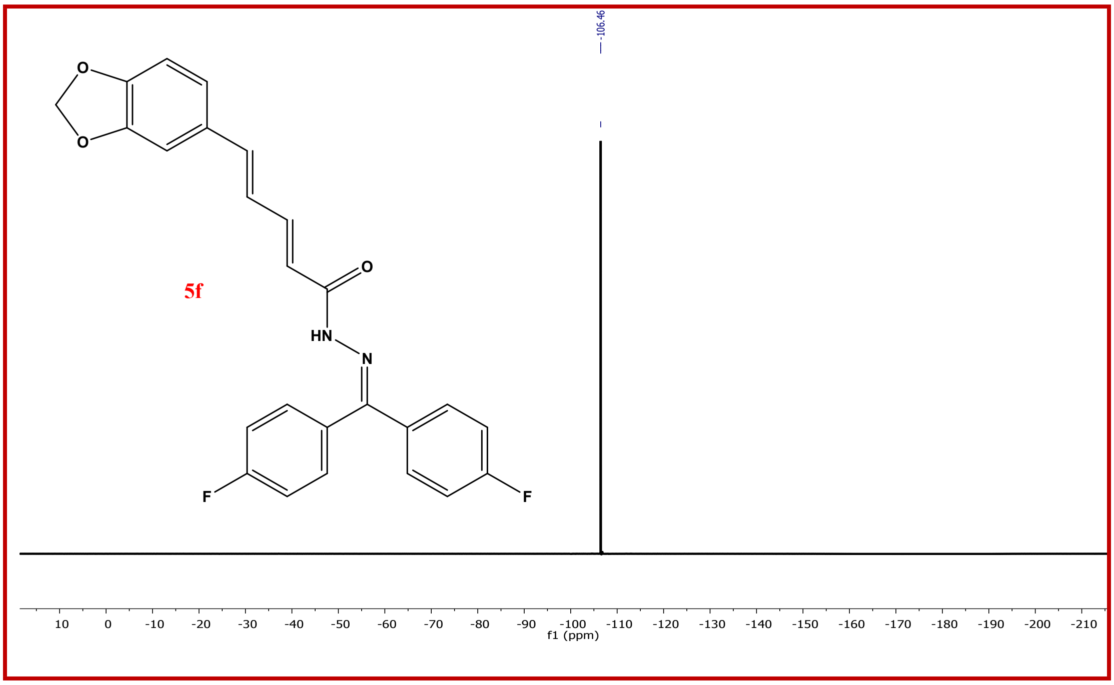
-106.46
5f
O
O
O
HN
N
F
F
10
0
-10
-20
-30
-40
-50
-60
-70
-80
-90
-100
-110
-120
-130
-140
-150
-160
-170
-180
-190
-200
-210
f1 (ppm)

Figure S7

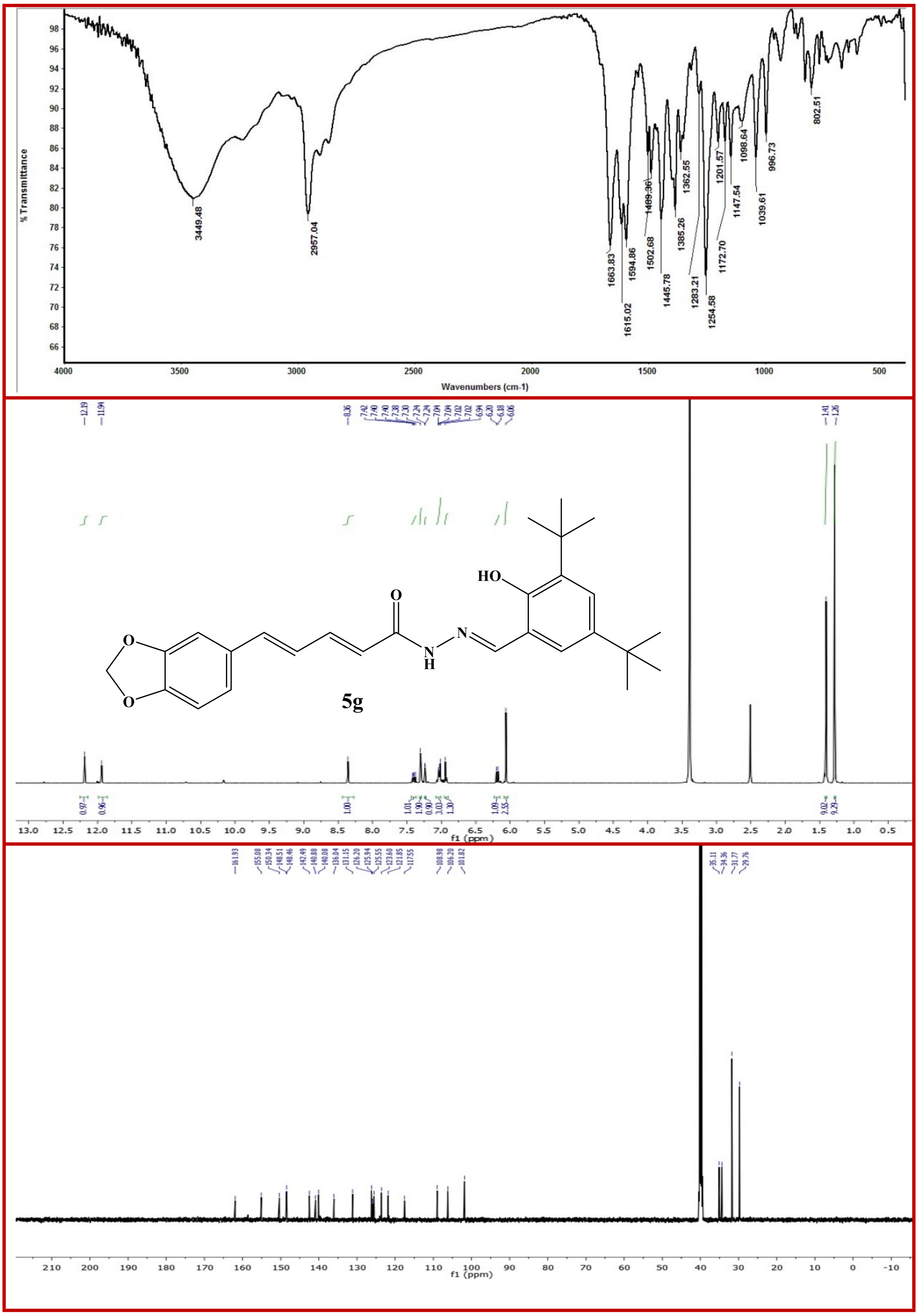

% Transmittance
Wavenumbers (cm-1)
3449.48
2957.04
1663.83
1615.02
1594.86
1502.68
1489.36
1445.78
1385.26
1362.55
1283.21
1254.58
1201.57
1172.70
1147.54
1098.64
1039.61
996.73
802.51
12.19
11.94
8.36
7.42
7.40
7.40
7.38
7.30
7.24
7.24
7.04
7.04
7.02
7.02
6.94
6.20
6.18
6.06
1.41
1.26
0.97
0.96
1.00
1.01
1.90
0.90
3.03
1.30
1.09
2.53
9.02
9.29
HO
O
N
H
O
O
5g
f1 (ppm)
161.93
155.08
150.34
148.51
148.46
142.49
140.88
140.08
136.04
131.15
126.20
125.94
125.55
123.60
121.85
117.55
108.98
106.20
101.82
35.11
34.36
31.77
29.76
f1 (ppm)

Figure S8

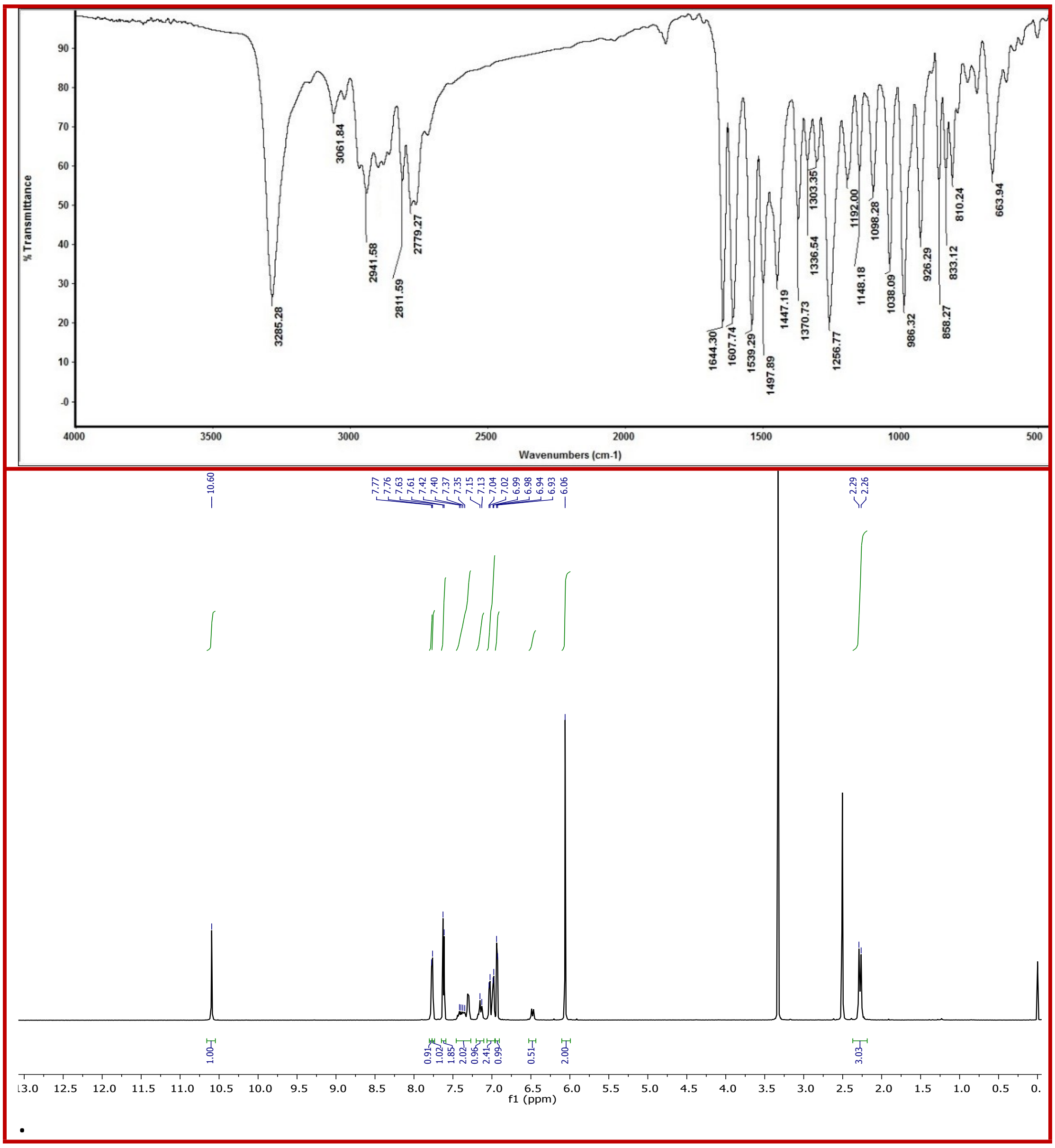

% Transmittance
Wavenumbers (cm-1)
3285.28
3061.84
2941.58
2811.59
2779.27
1644.30
1607.74
1539.29
1497.89
1447.19
1370.73
1336.54
1303.35
1256.77
1192.00
1148.18
1098.28
1038.09
986.32
926.29
858.27
833.12
810.24
663.94
10.60
7.77 7.76 7.63 7.61 7.42 7.40 7.37 7.35 7.15 7.13 7.04 7.02 6.99 6.98 6.94 6.93
6.06
2.29 2.26
1.00
0.91 1.02 1.85 2.02 0.96 2.41 0.99
0.51
2.00
3.03
f1 (ppm)

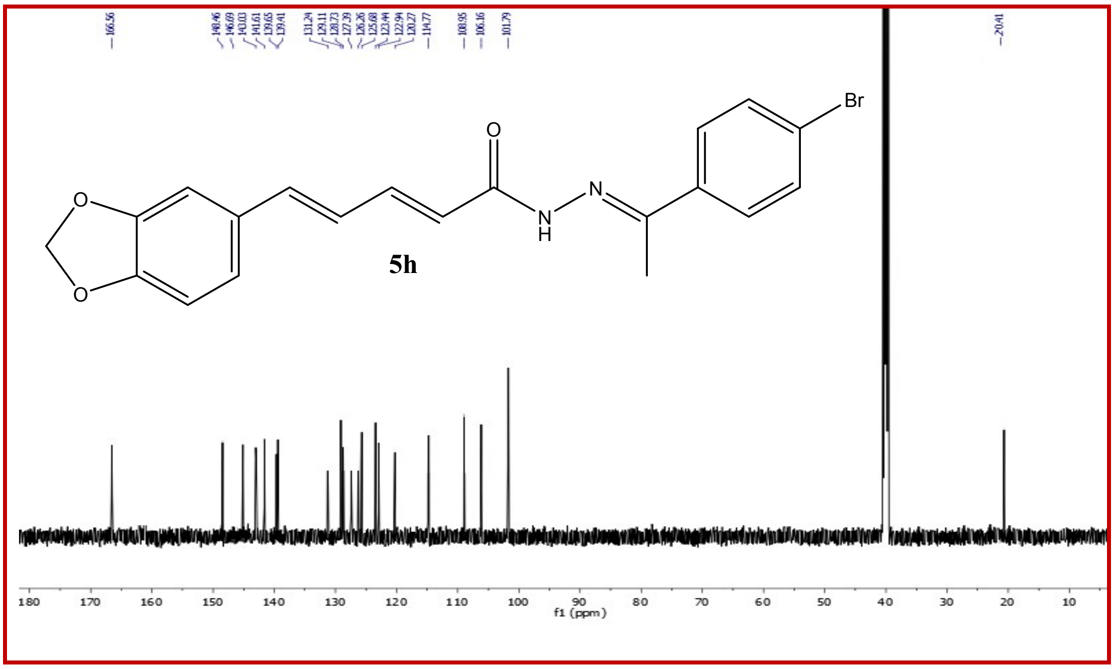
166.56
148.46
146.69
143.03
141.61
139.65
139.41
131.24
129.11
128.73
127.39
126.26
125.68
123.44
122.94
120.27
114.77
108.95
106.16
101.79
20.41
O
O
O
N
H
N
Br
5h
180 170 160 150 140 130 120 110 100 90 80 70 60 50 40 30 20 10
f1 (ppm)

Figure S9

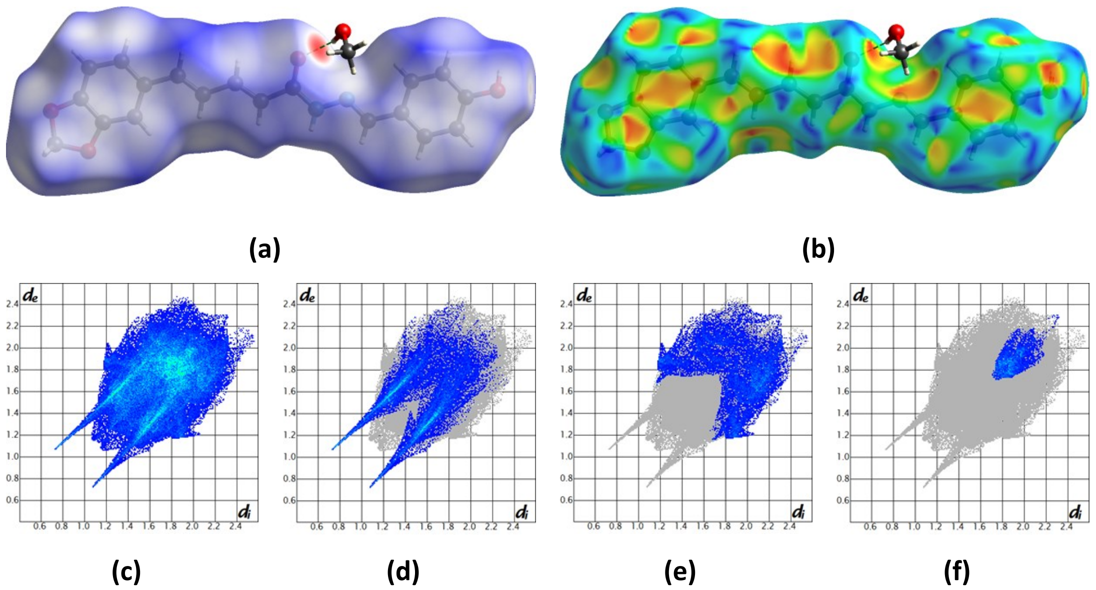

Figure S10

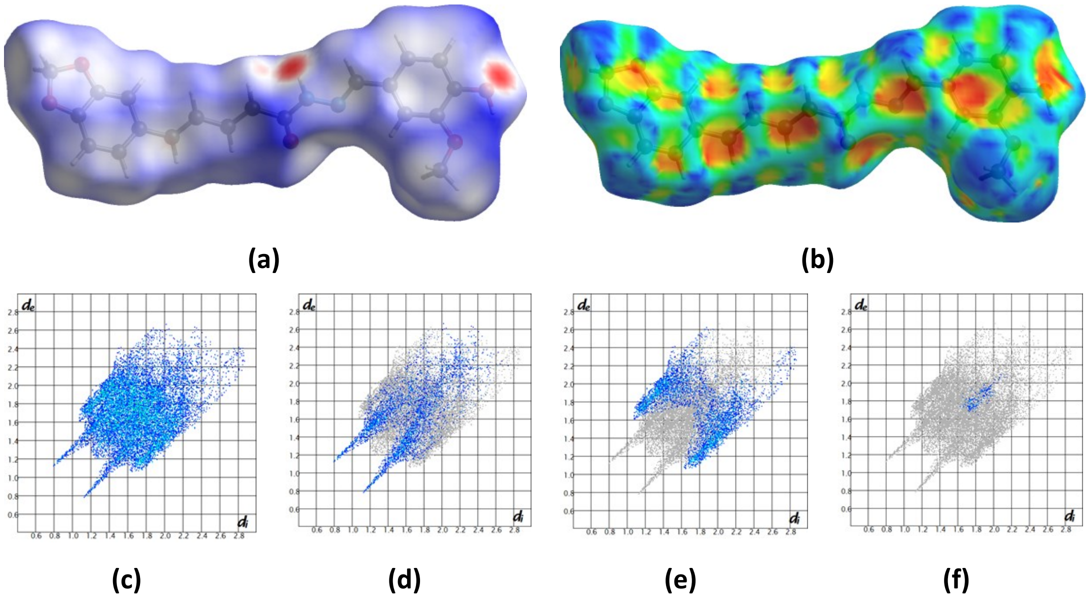

Figure S11

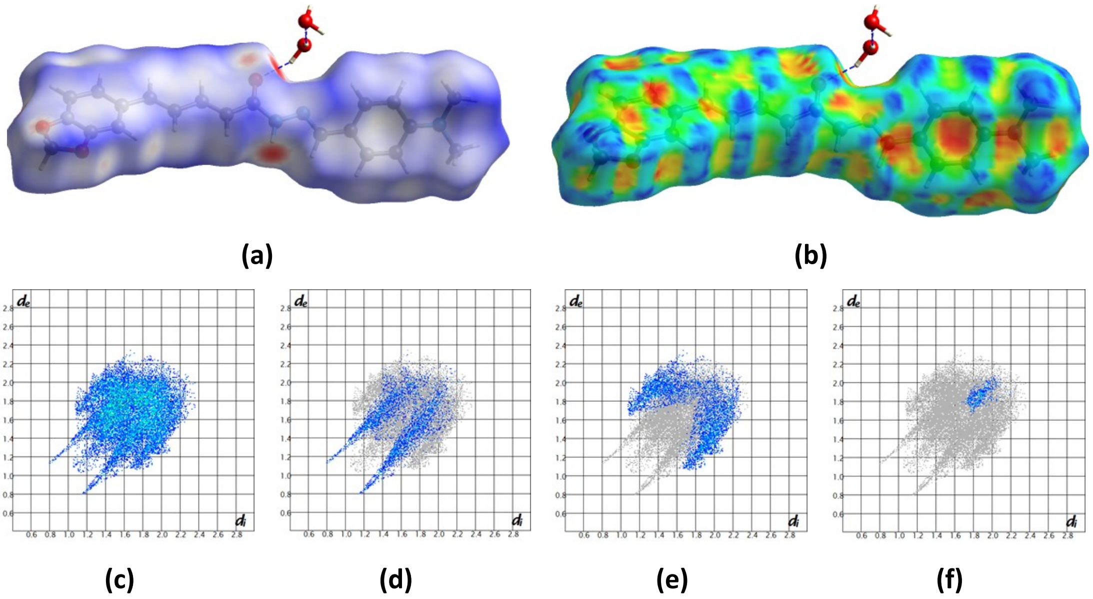

Figure S12

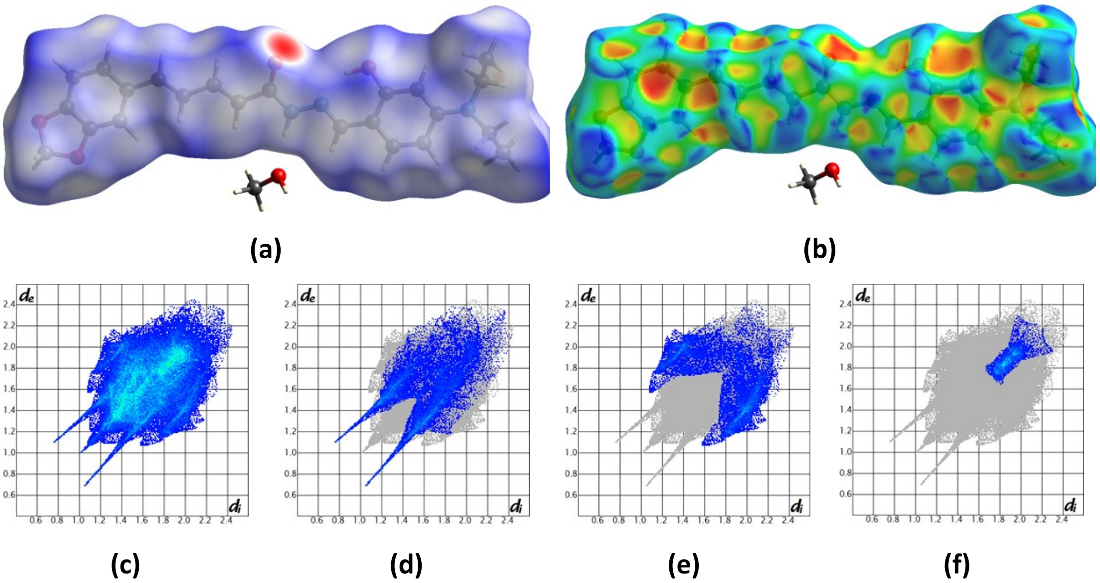

Figure S13

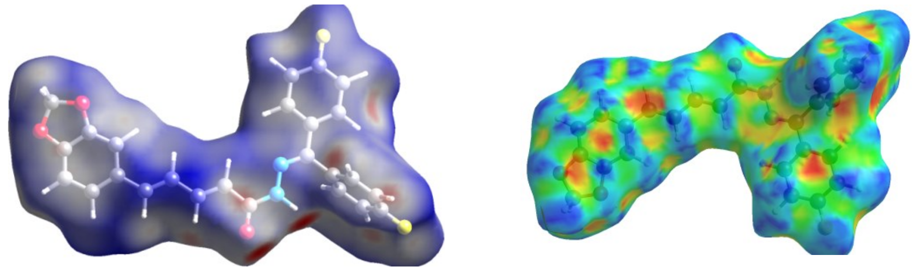

(a) (b)

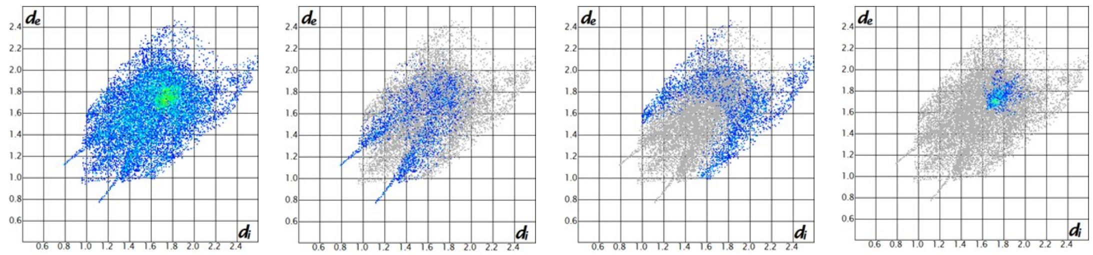

(c) (d) (e) (f)

Table S1

	5a	5b	5c	5d	5e	5f
Empirical formula	$C_{20}H_{20}N_2O_5$	$C_{20}H_{20}N_2O_6$	$C_{20}H_{18}N_2O_5$	$C_{21}H_{24.75}N_3O_{4.42}$	$C_{24}H_{29}N_3O_5$	$C_{25}H_{18}F_2N_2O_3$
Formula weight	368.38	384.38	366.36	389.87	439.50	432.41
Crystal system	Monoclinic	Monoclinic	Monoclinic	Monoclinic	Monoclinic	Triclinic
Space group	$P2_1/c$	$P2_1/c$	$P2_1/c$	$P2_1/c$	$P2_1/c$	*P-1*
Unit cell dimensions						
a (Å)	10.9249(5)	6.6192(6)	18.1988(7)	13.6957(16)	11.298(3)	5.329(3)
b (Å)	6.5740(3)	19.7039(17)	4.8105(2)	6.3687(7)	13.398(3)	10.708(5)
c (Å)	25.7341(11)	14.2723(13)	22.1672(8)	24.058(3)	15.678(4)	15.496(7)
α (°)	90	90	90	90	90	83.878(9)
β (°)	94.790(3)	99.079(5)	112.874(3)	104.090(6)	107.247(4)	87.608(9)
γ (°)	90	90	90	90	90	82.240(9)
Volume ($Å^3$)	1841.78(14)	1838.1(3)	1788.03(13)	2035.3(4)	2266.5(10)	870.8(7)
Z	4	4	4	4	4	4
Calc. density (Mg/m^3)	1.329	1.389	1.361	1.272	1.288	1.649
F(000)	776	808	768	828.0	936	448
Crystal size (mm)	0.12 x 0.1 x 0.1	0.13 x 0.12 x 0.1	0.12 x 0.1 x 0.1	0.12 × 0.04 × 0.03	0.15 x 0.15 x 0.1	0.20×0.12×0.10
θ range (°)	3.447 - 64.989	3.856 - 64.992	2.635 - 65.921	6.808-115.404	1.887 - 26.372	2.23 -25.00
Reflections collected	10352	10609	14927	9153	17545	6085
Independent reflections	3035	2973	3069	2692	4630	3045
Number of parameters/R_{int}	248 /0.0865	258/0.0552	247/0.0601	273/0.0786	295/0.0604	290/0.0520
Goof %	1.026	1.074	1.072	1.029	1.037	1.052
Final *R* indices [$I > 2\sigma$(I)]	$R1 = 0.0925$ $wR2 = 0.2368$	$R_1 = 0.0461$ $wR_2 = 0.1239$	R_1=0.0490 wR_2=0.1431	R_1 =0.0706 wR_2 =0.1880	R_1 = 0.0475 wR_2 =0.1263	R_1 = 0.0623 wR_2 = 0.1690
R indices (all data)	$R1 = 0.1011$ $wR2 = 0.2530$	R_1 =0.0507 wR_2 =0.1332	R_1=0.0687 wR_2=0.1575	R_1 =0.1081 wR_2 =0.2150	R_1 = 0.0948 wR_2 = 0.1535	R_1 = 0.0965 wR_2 = 0.1941
Max. diff. peak and hole ($eÅ^{-3}$)	*0.332, -0.362*	0.285, -0.225	0.215, -0.171	0.23, -0.19	0.257, -0.164	0.241, -0.241
